# Hepatoma‐Derived Growth Factor Coordinates STAT3 Pathway and Exosome‐Mediated Intrahepatic Crosstalk to Control Hepatic Steatosis and MASLD

**DOI:** 10.1002/advs.202523964

**Published:** 2026-05-01

**Authors:** Jian Wen, Dong Ding, Zengpeng Zheng, Xufeng Chen, Wenjing Li, Jiaxin Shen, Zhiwei Huang, Peng Tan, Junjie Bai, Xia Fang, Baofang Xing, Puyuan Hu, Linghao Xu, Haokai Yu, Yixi Wang, Zongzhe Jiang, Yang Long, Tiejun Zhou, Mingxin Ye, Yu Jiang, Aoyuan Cui, Hong Li, Qiurong Ding, Yong Xu, Yu Li, Weitong Su, Feng Shen, Chenlin Gao, Wenguang Fu

**Affiliations:** ^1^ Department of General Surgery (Hepatobiliary Surgery) Biliary‐Pancreatic Center The Affiliated Hospital Southwest Medical University Luzhou Sichuan China; ^2^ Metabolic Hepatobiliary and Pancreatic Diseases Key Laboratory of Luzhou City Academician (Expert) Workstation of Sichuan Province The Affiliated Hospital Southwest Medical University Luzhou Sichuan China; ^3^ Shanghai Institute of Nutrition and Health University of Chinese Academy of Sciences Chinese Academy of Sciences Shanghai China; ^4^ Department of Endocrinology and Metabolism Metabolic Vascular Disease Key Laboratory of Sichuan Province The Affiliated Hospital of Southwest Medical University Luzhou Sichuan China; ^5^ State Key Laboratory of Food Nutrition and Safety College of Biotechnology Tianjin University of Science and Technology Tianjin China; ^6^ Experimental Medicine Center Department of Endocrinology and Metabolism Metabolic Vascular Disease Key Laboratory of Sichuan Province The Affiliated Hospital of Southwest Medical University Luzhou Sichuan China; ^7^ Department of Pathology The Affiliated Hospital Southwest Medical University Luzhou Sichuan China; ^8^ Department of Gastroenterology & Endoscopy Xinhua Hospital Affiliated to Shanghai Jiao Tong University School of Medicine Shanghai China

**Keywords:** exosome, hepatic lipogenesis, hepatoma‐derived growth factor, MASLD, STAT3

## Abstract

Metabolic dysfunction‐associated steatotic liver disease has become a predominant cause of chronic liver disease worldwide and represents a major clinical management challenge owing to the scarcity of effective therapeutic interventions. However, the molecular mechanisms driving MASLD progression remain incompletely understood. Here, we identify hepatoma‐derived growth factor (HDGF) as a key regulator that integrates lipogenesis with intrahepatic inflammation in MASLD pathogenesis. Hepatic HDGF deficiency profoundly protects mice from high‐fat, high‐sucrose diet‐induced hepatic steatosis and inflammation. Mechanistically, HDGF promotes lipogenesis and hepatic steatosis by facilitating S6K1‐dependent phosphorylation of STAT3 at Ser727. Consistently, pharmacological inhibition of STAT3 by S3I‐201 abolishes HDGF‐induced lipogenic gene expression and hepatic steatosis in mouse models. Importantly, phosphorylation of HDGF at Ser165 is essential for its exosomal secretion from hepatocytes, thereby triggering proinflammatory macrophage activation. In humans, both serum and hepatic levels of HDGF are elevated and positively correlated with MASLD progression. Together, these findings uncover a mechanism that couples hepatic lipogenesis to intrahepatic macrophage activation, driving both steatosis and inflammation in MASLD. Targeting the HDGF‐STAT3 pathway and exosomal HDGF secretion may represent a potential therapeutic strategy for ameliorating metabolic dysfunction and hepatic inflammation in MASLD and related disorders.

## Graphical abstract

Hepatic HDGF as a key mediator in coordinating hepatic steatosis and intrahepatic crosstalk in MASLD. Activation of HDGF facilitates its interaction with both STAT3 and S6K1, driving the S6K1‐dependent STAT3 phosphorylation and subsequently enhancing hepatic lipogenesis. Furthermore, increased production and secretion of HDGF‐enriched exosomes trigger the intercellular macrophage proinflammatory response. Pharmacological or nanoparticle‐based neutralization approaches targeting HDGF‐STAT3 pathway may represent a promising therapeutic strategy for MASLD.

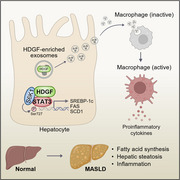

## 1 Introduction

Metabolic dysfunction‐associated steatotic liver disease (MASLD), formerly known as nonalcoholic fatty liver disease (NAFLD) [1], is a leading cause of chronic liver disease worldwide. MASLD may progress to its more severe form, metabolic dysfunction‐associated steatohepatitis (MASH), leading to liver cirrhosis, hepatocellular carcinoma, and end‐stage liver disease [2, 3]. As the hepatic manifestation of the metabolic syndrome, MASLD is frequently associated with obesity, dyslipidemia, and type 2 diabetes [4, 5]. Given the absence of effective clinical therapies, elucidating the pathogenesis and identifying potential therapeutic targets for MASLD are of critical importance.

Hepatic steatosis is driven by metabolic alterations that promote intrahepatic lipid accumulation, largely through enhanced de novo lipogenesis (DNL) mediated by the transcription factor sterol regulatory element‐binding protein 1c (SREBP‐1c) [6, 7]. The mechanistic target of rapamycin complex 1 (mTORC1) signaling activates SREBP‐1c via an S6K1‐mechanism [8, 9]. As a pivotal transcription factor, signal transducer and activator of transcription 3 (STAT3) integrates cytokines and growth hormone signals to direct diverse processes, including proliferation, inflammatory responses, and the regulation of glucose and lipid metabolism in tumor cells [10, 11]. However, the mechanism by which STAT3 regulates lipid synthesis in MASLD progression remains elusive.

Intrahepatic crosstalk between hepatocytes, hepatic stellate cells, and immune cells plays critical roles in the pathogenesis of MASLD [12, 13, 14], mediated by direct cellcell interactions, soluble factors, and extracellular vesicles [15, 16]. Recently, the fibroblast growth factor 21 (FGF21), a hormone primarily derived from hepatocytes, has emerged as a key mediator in limiting liver inflammation and injury via reprogramming intrahepatic cell functions [17, 18, 19]. Furthermore, the extracellular matrix protein osteopontin (OPN) facilitates a pro‐fibrogenic crosstalk among hepatocytes, hepatic stellate cells, and biliary epithelial cells, thereby fueling the progression of MASH [20, 21, 22, 23]. Nevertheless, the precise mechanisms governing intrahepatic crosstalk during MASLD remain incompletely understood.

Hepatoma‐derived growth factor (HDGF) is identified as a hepatocyte‐derived factor involved in cell proliferation, apoptosis, and angiogenesis [24, 25]. Notably, HDGF interacts with hepatoma‐derived growth factor‐related protein 2 (HRP2) and is secreted upon cellular stress, suggesting a potential role in mediating metabolic adaptation [26, 27, 28]. However, whether and how HDGF regulates MASLD progression remains largely unknown. Here, we identify HDGF as a crucial mediator that couples hepatic steatosis with intrahepatic inflammation during MASLD progression. The in vivo and in vitro investigations demonstrate that (1) hepatic HDGF deficiency attenuates hepatic steatosis and metabolic dysfunction; (2) HDGF stimulates phosphorylation of STAT3 at Ser727 and enhances hepatic lipogenesis by the recruitment and activation of S6K1; (3) hepatocyte‐derived exosomal HDGF triggers proinflammatory macrophage activation in the liver; (4) inhibition of STAT3 attenuates HDGF‐exacerbated hepatic lipogenesis and MASLD pathogenesis.

## 2 Materials and Methods

### 2.1 Human Liver Specimens

The MASLD and nondiabetic control liver specimens were obtained between November 2023 and June 2024 from the Affiliated Hospital of Southwest Medical University. Study of these specimens was approved by the Ethics Committee of the Affiliated Hospital of Southwest Medical University (No. KY2022167), and was conducted in accordance with the 1975 Declaration of Helsinki.

### 2.2 Animal Model and Diets

Hepatocyte‐specific HDGF knockout mice were generated by tail vein injection of AAV8‐TBG‐Cre‐sgHDGF (1 × 10^11^ vg/mouse) into adult Credependent Cas9 knockin mice (Rosa^Cas9+/−^) mice. Control mice received AAV8‐TBG‐Cre‐sgGFP at an equivalent titer. Mice were fed a high‐fat, high‐sucrose (HFHS) diet (D12327, Research Diets) for 16 weeks. All mice were housed under a 12:12‐hour light/dark cycle at a controlled temperature. All animal experimental protocols were approved by the Institutional Animal Care and Use Committee at the Shanghai Institute of Nutrition and Health, Chinese Academy of Sciences (Approval No. SINH‐2025‐LY‐1).

### 2.3 Generation of HDGF Knockout Cell Lines by CRISPR‐Cas9

HDGF knockout Huh7 cell lines were generated using the LentiCRISPRv2 system (#52961, Addgene). sgRNAs targeting HDGF were designed using the Zhang Lab CRISPR design tool and cloned into LentiCRISPRv2. Transfected cells underwent puromycin selection (2 µg/mL, 48 h), followed by single‐cell sorting into 96‐well plates using limiting dilution. After 14 days expansion, monoclonal colonies were screened by immunoblotting for HDGF protein depletion and validated by Sanger sequencing of the target locus.

### 2.4 Statistical Analysis

Statistical analysis was performed using GraphPad Prism 8.0 software. Data were tested for normality and homogeneity of variance before formal analysis. Data were presented as mean ± SEM. Sample size (n) is indicated in each figure legend. For clinical sample comparisons of gene expression levels, an unpaired two‐tailed Student's *t*‐test was used. Pearson correlation analysis was performed to evaluate clinical data correlations. For animal and in vitro studies, statistical significance was evaluated using the unpaired two‐tailed Student's *t*‐test for two groups. For more than two groups, data were analyzed by one‐way ANOVA or two‐way ANOVA. A *p*‐value < 0.05 was considered statistically significant.

## 3 Results

### 3.1 Elevation of Hepatic HDGF is Associated With MASLD Severity

**FIGURE 1:**
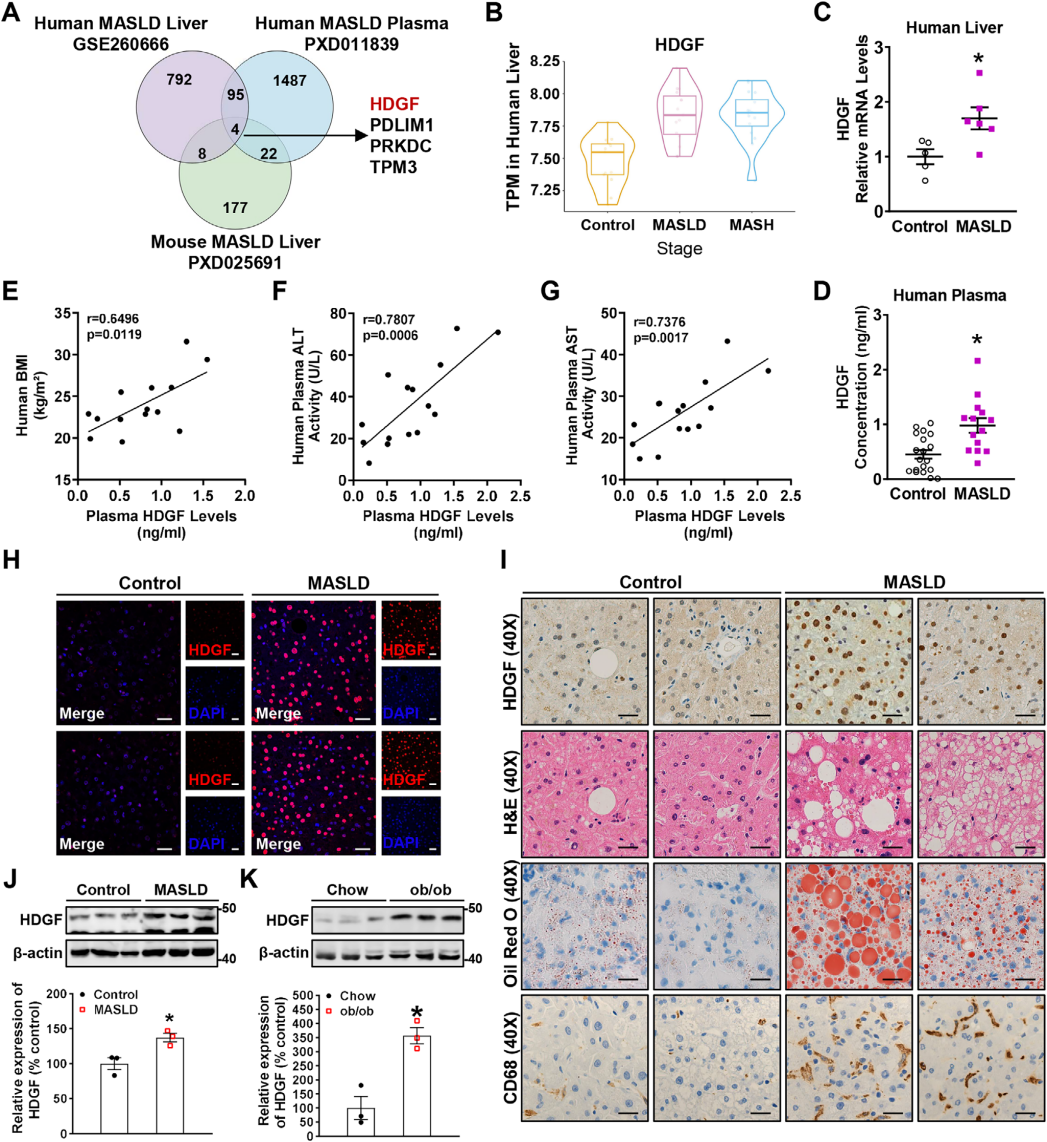
Hepatic HDGF is increased and positively correlated with MASLD progression in humans. (A) Venn diagram showing differentially expressed genes and proteins in the liver and plasma of humans and mice. (B) Expression levels of HDGF are increased in livers of control, MASLD, and MASH subjects (GEO accession GSE135251). Thick line: median; dotted lines: interquartile range (IQR); width: data density. (C) mRNA levels of HDGF in human liver tissues. n = 5–6. (D) Serum HDGF levels measured by ELISA. n = 14–19. (E–G) Correlation of plasma HDGF levels with BMI (E), ALT (F), and AST levels (G) in humans. n = 14–15. Pearson correlation analysis. (H) The expression of HDGF in human livers was analyzed by immunofluorescence. Representative images were shown. n = 3. Scale bar, 50 µm. (I) Representative HDGF immunohistochemical staining, H&E staining, Oil red O staining, and CD68 immunohistochemical staining in the liver sections of control and MASLD humans. n = 3. Scale bar, 50 µm. (J,K) The protein levels of HDGF were increased in liver from MASLD humans (J) and ob/ob mice (K). Lysates from humans and mice livers were analyzed by immunoblots. Densitometric quantification of HDGF normalized to β‐actin. Data were presented as mean ± SEM. n = 3. *p < 0.05, unpaired two‐tailed Student's *t*‐test.

To identify the key factor underlying MASLD pathogenesis, an integrated proteomic analysis of human MASLD liver tissues, paired serum samples, and murine models was performed, and a total of 4 candidate proteins were identified. Among the top candidate proteins, the hepatoma‐derived growth factor (HDGF) was distinguished by its consistent upregulation across all human and murine datasets and by its annotation as a secretory protein in the UniProt database (Figure 1A). Interestingly, hepatic HDGF expression was significantly elevated in MASLD patients, suggesting its potential involvement in regulating hepatocyte metabolic pathways (Figure 1B,C; Figure S1A–C). Notably, the circulating HDGF levels were increased and showed positive correlations with pivotal clinical indices, including BMI and plasma levels of triglyceride, cholesterol, alanine aminotransferase, and aspartate aminotransferase (Figure 1D–G; Figure S1D,E). Moreover, the upregulated levels of hepatic HDGF were also verified by immunohistochemical, immunofluorescence staining, and immunoblotting, which is consistent with the observation of severe hepatic lipid accumulation and inflammation under MASLD pathological conditions (Figure 1H–K; Figure S1F–H). Immunofluorescence analysis showed that HDGF was predominantly expressed in hepatocytes, but not in myeloid cells, in both human and mouse livers (Figure S1I). Collectively, these results suggest that hepatocyte HDGF is a potential regulator involved in MASLD progression.

### 3.2 Hepatocyte HDGF Regulates Hepatic Steatosis in MASLD Mice Fed With HFHS Diet

**FIGURE 2:**
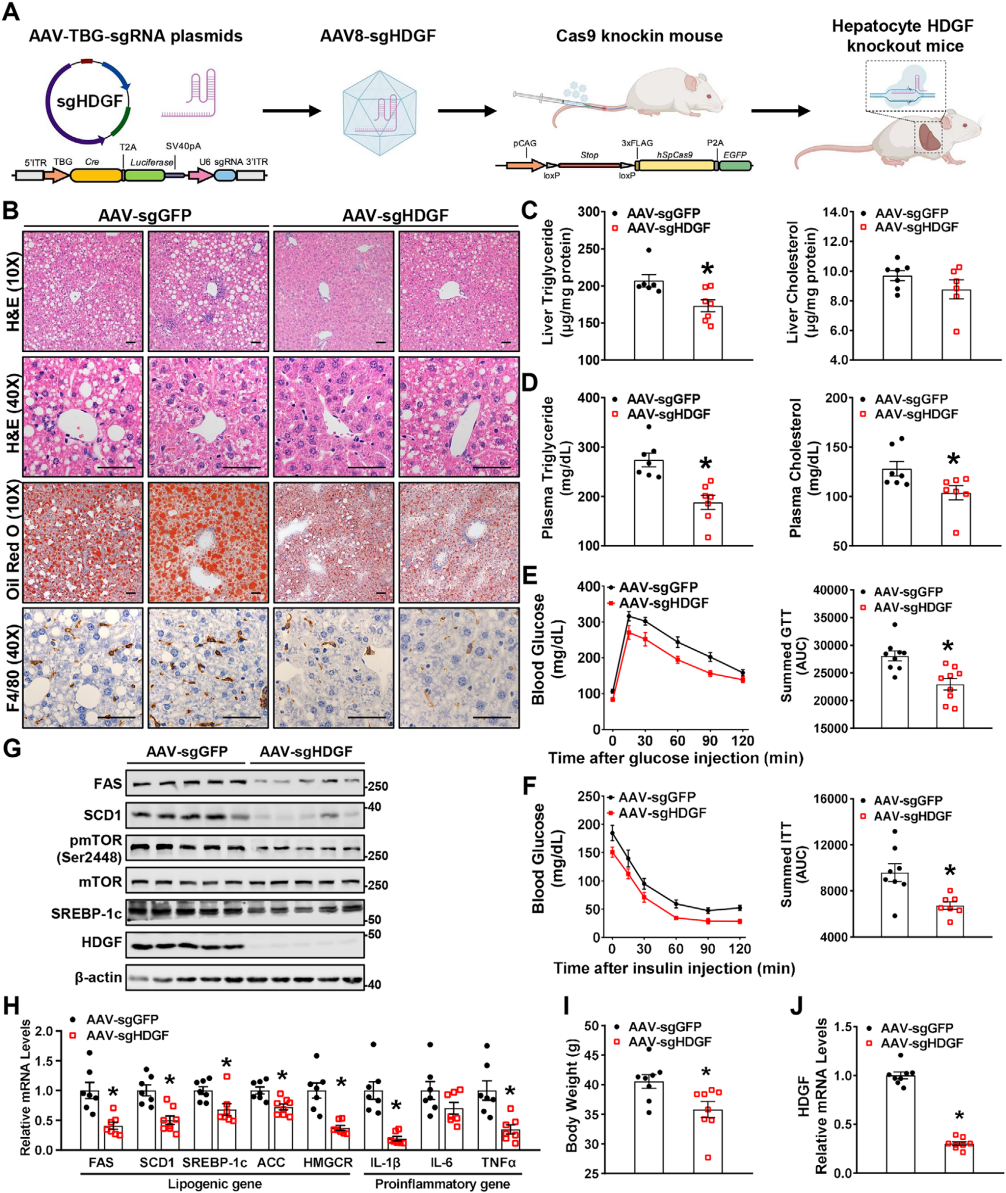
HDGF deficiency improves hepatic steatosis and metabolic disorders in mice fed with HFHS diet. Cas9‐knockin mice were injected with liver‐specific AAV‐TBG‐sgGFP or AAV‐TBG‐sgHDGF at 8 weeks of age, followed by 16 weeks HFHS‐diet feeding. (A) The schematic diagram of liver‐specific HDGF knockout mice. (B) Representative H&E staining, Oil red O staining, and F4/80 immunohistochemical staining in liver sections. Scale bar, 50 µm. (C) Liver triglyceride and cholesterol levels were assessed. (D) Plasma triglyceride and cholesterol levels were measured. n = 6–7. (E) Glucose tolerance tests and respective area under the curve (AUC). (F) Insulin tolerance test and respective area under the curve (AUC). n = 7–8. (G) Hepatic expression levels of lipid metabolism related protein were decreased in HDGF deficiency mice. Lysates were analyzed by immunoblots. (H) mRNA levels of lipogenic and proinflammatory genes were decreased in HDGF deficiency mice. (I) Body weight. n = 8. (J) HDGF knockout efficiency in liver measured by qPCR. Data were presented as mean ± SEM. n = 8. *p < 0.05, unpaired two‐tailed Student's *t*‐test.

To investigate the role of HDGF in MASLD pathogenesis, hepatocyte‐specific HDGF knockout mice were generated by delivering an HDGF‐targeting (AAV‐sgHDGF) or control (AAV‐sgGFP) adeno‐associated virus via tail vein injection to Cas9‐knockin mice (Figure 2A). Hepatocyte‐specific deletion of HDGF was successfully confirmed, but not in F4/80‐positive myeloid cells or desmin‐positive stellate cells (Figure 2J; Figure S2A). After 16 weeks of high‐fat, high‐sucrose (HFHS) diet feeding, hepatocyte‐specific HDGF knockout mice exhibited marked amelioration of hepatic steatosis, as evidenced by reduced lipid accumulation and inflammatory cell infiltration on liver histology (Figure 2B; Figure S2B), as well as lower hepatic triglyceride and cholesterol content (Figure 2C). As shown in Figure 2D, the plasma triglyceride and cholesterol levels were also significantly reduced in HDGF‐deficient mice fed a HFHS diet compared to controls. Moreover, HDGF deficiency markedly alleviated HFHS diet‐induced systemic glucose intolerance and insulin resistance (Figure 2E,F). Notably, HDGF deletion caused a significant reduction in the expression of hepatic lipogenic and proinflammatory genes in the liver of HFHS diet‐fed mice (Figure 2G,H). In addition, hepatocyte‐specific HDGF‐deficient mice exhibited decreased body weight (Figure 2I). In contrast, AAV‐mediated HDGF hepatic overexpression induced a significant increase in hepatic steatosis and deteriorated glucose tolerance and insulin sensitivity (Figure S3A–F). Meanwhile, in primary hepatocytes stimulated with glucose and insulin, HDGF overexpression further promoted lipid accumulation (Figure S3G,H). These results demonstrate that HDGF plays a key role in the regulation of hepatic steatosis under MASLD pathogenesis.

### 3.3 HDGF Interacts With STAT3 and Enhances Its Transcriptional Activity

**FIGURE 3:**
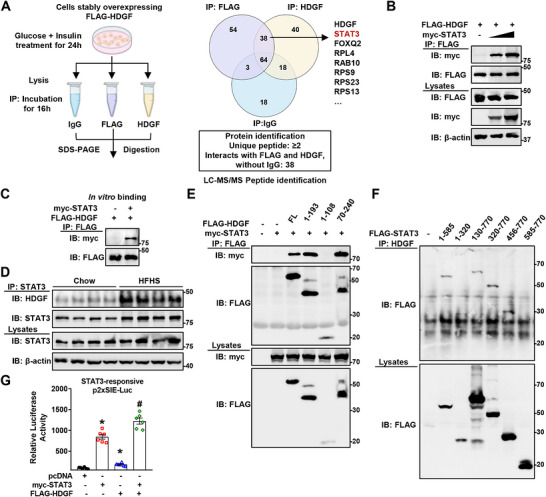
HDGF associates with STAT3. (A) Schematic workflow for mass spectrometry. Huh7 hepatocytes stably overexpressing FLAG‐HDGF were stimulated with 30 mm glucose and 100 nm insulin for 24 h, followed by immunoprecipitated with anti‐FLAG, anti‐HDGF, or IgG as negative control. Proteins interacting with antibodies were analyzed by mass spectrometry. Veen diagram identifies STAT3 as a binding protein of both HDGF and FLAG‐tag. (B) STAT3 associates with HDGF. HEK293T cells were transfected with FLAG‐tagged HDGF and myc‐tagged STAT3. Anti‐FLAG immunoprecipitants were analyzed by immunoblotting. (C) In vitro binding assay. myc‐tagged STAT3 and FLAG‐tagged HDGF proteins were overexpressed and purified from HEK293T cells, and then incubated in in vitro binding systems. Anti‐FLAG immunoprecipitants were analyzed by immunoblotting. (D) STAT3 showed interaction with HDGF in liver. Liver lysates from chow diet or 16 weeks HFHS‐fed mice were immunoprecipitated with STAT3 antibody and analyzed by immunoblotting. (E) Mapping of the STAT3‐binding region in HDGF. FLAG‐tagged HDGF and its truncated mutants were co‐transfected with myc‐tagged STAT3 in HEK293T cells as indicated. Anti‐FLAG immunoprecipitants were analyzed by immunoblotting. (F) Mapping of the HDGF‐binding region in STAT3. FLAG‐tagged STAT3 and its truncated mutants were co‐transfected in HEK293T cells. Anti‐HDGF immunoprecipitants were analyzed by immunoblotting. (G) HDGF potentiates transcriptional activity of STAT3. HEK293T cells were transfected with STAT3‐responsive p2×SIE‐Luc and Renilla luciferase reporter plasmid, together with HDGF, STAT3, or pcDNA as indicated. Dual luciferase activities were measured. Data were presented as mean ± SEM. n = 6. *p < 0.05 vs. pcDNA, #*p* < 0.05 vs. myc‐STAT3, one‐way ANOVA.

We next sought to identify the downstream mediator of HDGF in regulating hepatic lipogenesis. Protein‐protein interaction (PPI) proteomics in Huh7 hepatocytes identified 38 HDGF‐interacting proteins, among which STAT3, a key transcription factor, emerged as one of the top candidates (Figure 3A). The interaction between HDGF and STAT3 was confirmed by cellular co‐immunoprecipitation and in vitro binding assays using purified proteins (Figure 3B,C). Importantly, this endogenous interaction was detected in mouse liver and markedly enhanced in HFHS diet‐induced MASLD (Figure 3D). To delineate the regions responsible for the binding between HDGF and STAT3, we performed domain mapping assays using a series of truncation mutants of both proteins. These experiments revealed that the N‐terminal region of HDGF (amino acids 1–108) and C‐terminal transactivation domain of STAT3 (amino acids 585–770) were sufficient to direct their interaction (Figure 3E,F). To examine the effects of HDGF on the transcriptional activity of STAT3, we performed luciferase reporter assays in HEK293T cells transfected with a STAT3‐responsive reporter (p2×SIE‐Luc), along with myc‐STAT3 and either FLAG‐HDGF or an empty vector. As shown in Figure 3G, luciferase activity was induced by STAT3 and was further enhanced by co‐transfecting HDGF. Taken together, these results suggest that HDGF physically associates with STAT3 and thereby modulates its transcriptional activity.

### 3.4 Phosphorylation of STAT3 Mediates HDGF‐Induced Hepatic Lipogenesis

**FIGURE 4:**
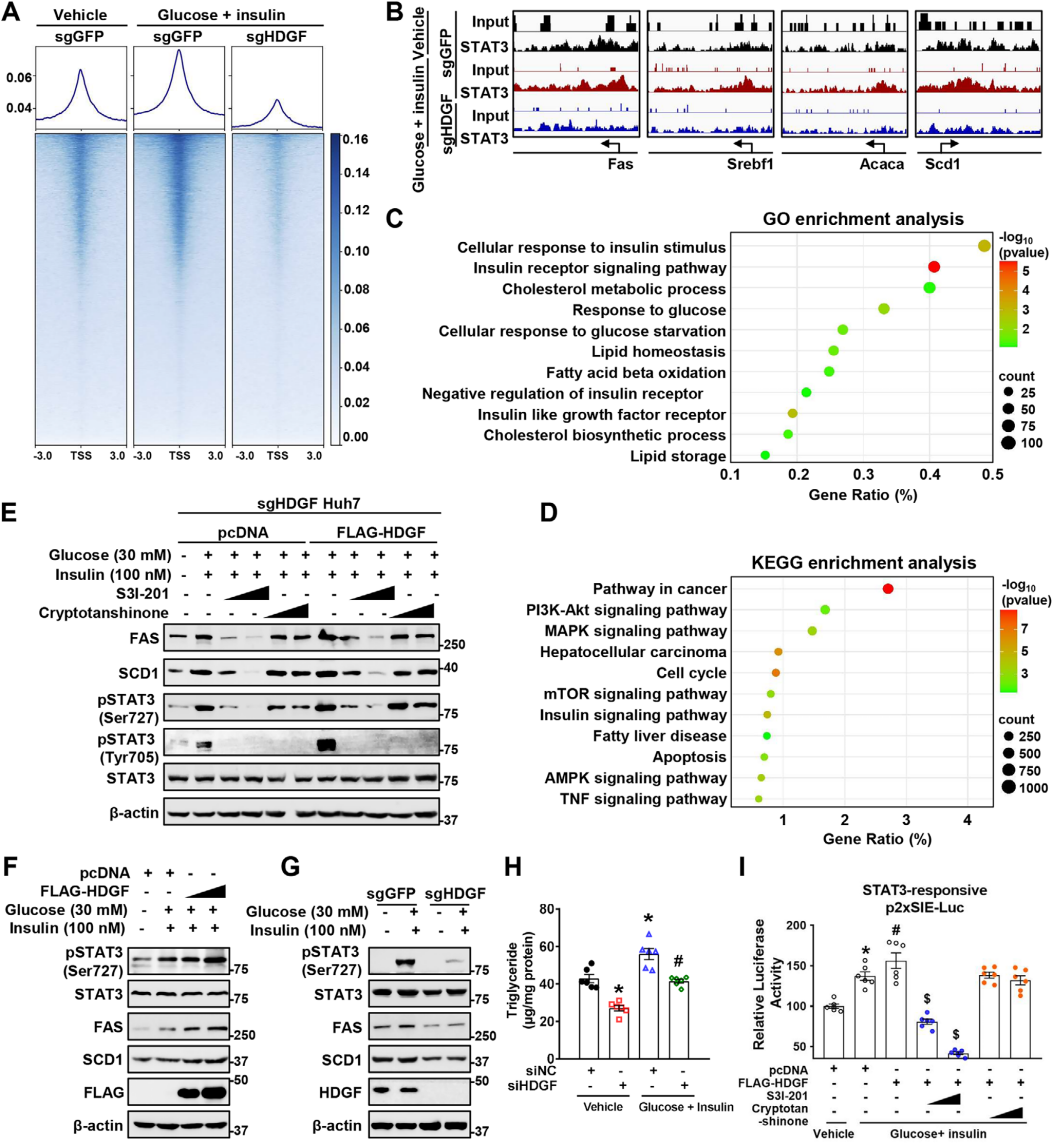
HDGF‐induced lipogenesis is mediated by STAT3‐dependent phosphorylation. (A–D) HDGF deficiency repressed the transcriptional activity of STAT3. sgRNA control (sgGFP) and HDGF‐deficient (sgHDGF) Huh7 hepatocytes were treated with 30 mm glucose and 100 nm insulin or vehicle for 24 h, followed by the STAT3 chromatin immunoprecipitation sequencing (ChIP‐seq). (A) Heatmaps showing density of mapped STAT3 ChIP‐seq reads from single biological replicates 3 kb up‐ and downstream of transcriptional start sites (TSS). (B) Integrative Genomics Viewer (IGV) plot showed the STAT3 binding on lipogenic genes locus. (C,D) GO enrichment analysis (C) and KEGG enrichment analysis (D) of the downstream differentially expressed genes of STAT3 repressed by HDGF deficiency. (E) STAT3 phosphorylation at Ser727 is required for HDGF‐induced lipogenic gene expression. HDGF‐deficient (sgHDGF) Huh7 cells were transfected with or without FLAG‐HDGF, followed by treatment with 30 mm glucose and 100 nm insulin for 12 h. Subsequently, cells were treated with the pan‐STAT3 inhibitor S3I‐201 (50, 100 nm) or the pSTAT3 (Tyr705)‐specific inhibitor cryptotanshinone (CPT, 10, 20 µm) for an additional 12 h. Lysates were analyzed by immunoblots. (F,G) HDGF is essential for glucose and insulin‐induced STAT3 phosphorylation at Ser727 and lipogenic gene expression. (F) Huh7 cells were transfected with FLAG‐HDGF, followed by treatment with 30 mm glucose and 100 nm insulin for 24 h. (G) sgRNA control (sgGFP) and HDGF‐deficient (sgHDGF) Huh7 cells were treated with 30 mm glucose and 100 nm insulin for 24 h. Lysates were analyzed by immunoblots. (H) Triglyceride content was measured in primary hepatocytes. Primary hepatocytes were transfected with siRNA, then treated with 30 mm glucose and 100 nm insulin for 24 h. Triglyceride content was measured and adjusted by protein content. Data were presented as mean ± SEM. n = 6. *p < 0.05 vs. siNC and PBS, #*p* < 0.05 vs. siHDGF and PBS, one‐way ANOVA. (I) The luciferase activities of STAT3‐Luc in HEK293T cells. HEK293T cells were transfected with STAT3‐responsive p2×SIE‐Luc reporter and Renilla luciferase reporter plasmid pRL‐SV40, together with pcDNA and FLAG‐HDGF as indicated, and then treated with 30 mm glucose and 100 nm insulin for 12 h. Subsequently, cells were treated with the pan‐STAT3 inhibitor S3I‐201 (50, 100 nm) or the pSTAT3 (Tyr705)‐specific inhibitor cryptotanshinone (CPT, 10, 20 µm) for an additional 12 h. Dual luciferase activities were measured. Data were presented as mean ± SEM. n = 6. *p < 0.05 vs. pcDNA and vehicle, #*p* < 0.05 vs. pcDNA with glucose and insulin, $*p* < 0.05 vs. FLAG‐HDGF with glucose and insulin, one‐way ANOVA.

To further assess the roles of HDGF in regulating STAT3 transcriptional activity and downstream target gene expression, the STAT3 chromatin immunoprecipitation sequencing (ChIP‐seq) was performed in hepatocytes. Notably, glucose and insulin treatment promoted the recruitment of STAT3 to lipogenic gene promoters; these effects were abolished by HDGF deletion in hepatocytes (Figure 4A,B; Figure S4A–E). Gene Ontology (GO) and Kyoto Encyclopedia of Genes and Genomes (KEGG) analysis revealed that STAT3 target genes involved in metabolic pathways were downregulated by HDGF deficiency (Figure 4C,D). These results demonstrate that HDGF deficiency reduces the transcriptional activity of STAT3 and its downstream target lipogenic gene expression.

Next, we investigated the mechanism by which HDGF regulates lipogenesis via STAT3. Previous studies have shown that phosphorylation of STAT3 is required for its dimerization, nuclear translocation, and transcriptional regulation of downstream target genes [29]. Glucose and insulin elevated lipogenic gene expression and STAT3 phosphorylation, and these effects were further enhanced by HDGF repletion. Importantly, HDGF‐induced lipogenic responses and STAT3 phosphorylation were specifically abrogated by the dual STAT3 inhibitor S3I‐201, but not by the Tyr705‐specific inhibitor cryptotanshinone (Figure 4E), suggesting that phosphorylation of STAT3 at Ser727 was required for HDGF‐induced hepatic lipogenesis. Moreover, glucose and insulin‐induced phosphorylation of STAT3 at Ser727 was further augmented by HDGF overexpression and abolished by HDGF deficiency (Figure 4F,G). Accordingly, HDGF knockdown in primary hepatocytes significantly reduced intracellular lipid accumulation under glucose and insulin stimulation (Figure 4H). Furthermore, S3I‐201 inhibited the HDGF‐induced enhancement of STAT3 transcriptional activity upon co‐treatment with glucose and insulin (Figure 4I). Importantly, both S3I‐201 and cryptotanshinone inhibited STAT3 luciferase activity stimulated by IL‐6 (Figure S4F), suggesting that phosphorylation of STAT3 at Tyr705 is sufficient to mediate its activation by inflammatory signals. These findings suggest that HDGF‐promoted hepatic lipogenesis specifically requires phosphorylation of STAT3 at Ser727, while phosphorylation at Tyr705 is dispensable for this process.

### 3.5 HDGF Promotes the Phosphorylation of STAT3 in an S6K1‐Dependent Manner

**FIGURE 5:**
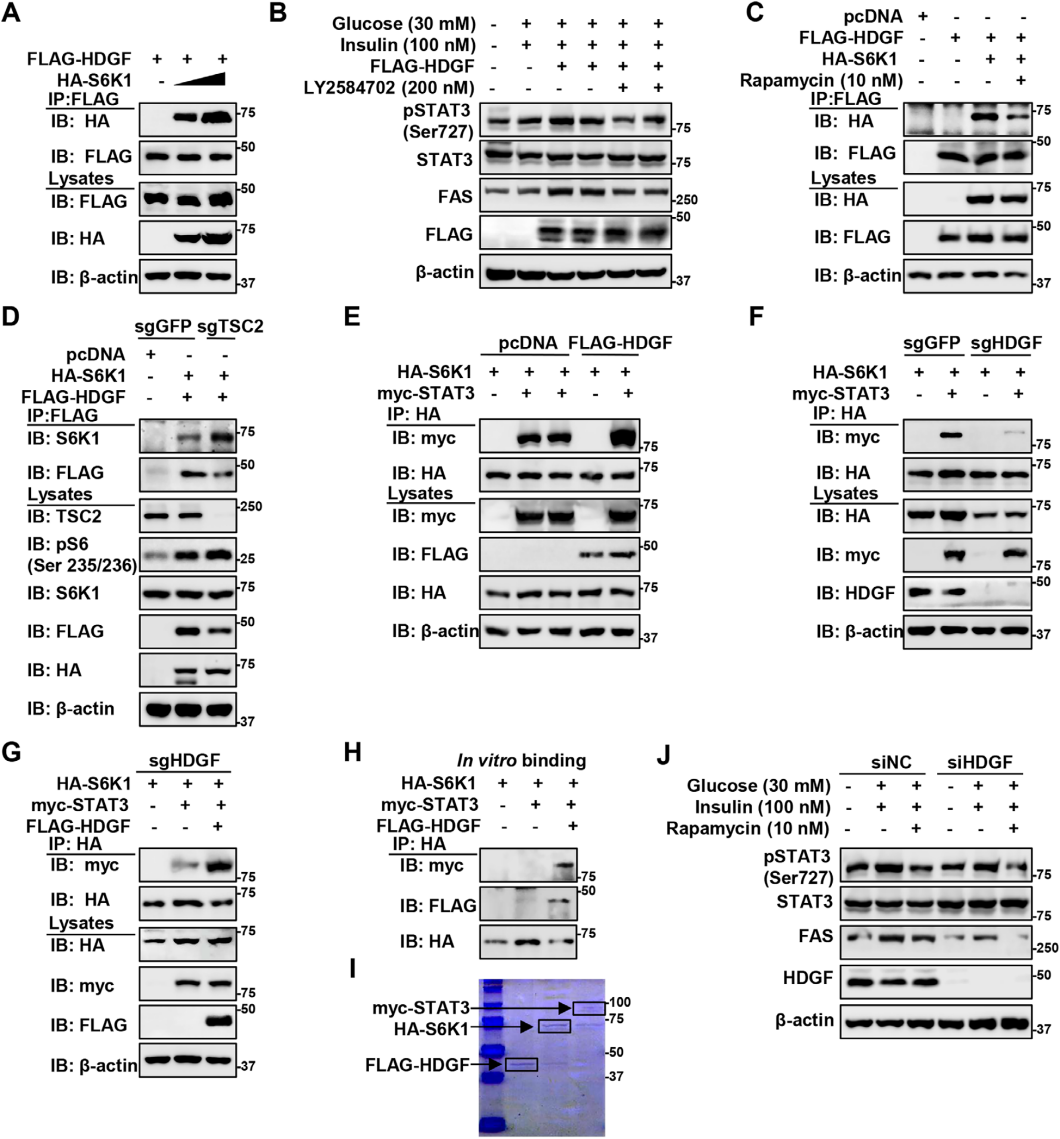
S6K1 is required for the HDGF‐induced STAT3 phosphorylation and lipogenesis. (A) S6K1 interacts with HDGF. HEK293T cells were transfected with FLAG‐HDGF or HA‐tagged S6K1. Anti‐FLAG immunoprecipitants were analyzed by immunoblotting. (B) Inhibition of S6K1 decreased phosphorylation levels of STAT3. Primary hepatocytes were transfected with FLAG‐HDGF and treated with 30 mm glucose and 100 nm insulin for 12 h, followed by treatment with LY2584702 for another 12 h. Lysates were analyzed by immunoblots. (C) Inhibition of mTOR decreased the interaction between HDGF and S6K1. Huh7 cells were transfected with FLAG‐HDGF and HA‐S6K1, then treated with 10 nm rapamycin for 24 h. Anti‐FLAG immunoprecipitants were analyzed by immunoblotting. (D) TSC2 deficiency increased the association between HDGF and S6K1. sgRNA control (sgGFP) and TSC2‐deficient (sgTSC2) Huh7 cells were transfected with FLAG‐HDGF and HA‐S6K1. Anti‐FLAG immunoprecipitants were analyzed by immunoblotting. (E–G) The association between S6K1 and STAT3 is dependent on HDGF. (E) HDGF facilitated the interaction between S6K1 and STAT3. HA‐S6K1 were co‐transfected with myc‐STAT3 and FLAG‐HDGF in Huh7 cells. Anti‐HA immunoprecipitants were analyzed by immunoblotting. (F) HDGF deletion reduced the interaction between S6K1 and STAT3. sgRNA control (sgGFP) and HDGF‐deficient (sgHDGF) Huh7 cells were transfected with HA‐S6K1 and myc‐STAT3. Anti‐HA immunoprecipitants were analyzed by immunoblotting. (G) HDGF rescued the binding of S6K1 to STAT3. HDGF‐deficient (sgHDGF) Huh7 cells were transfected with HA‐S6K1, myc‐STAT3, and FLAG‐HDGF. Anti‐HA immunoprecipitants were analyzed by immunoblotting. (H,I) In vitro binding assay. HA‐tagged S6K1, myc‐tagged STAT3, and FLAG‐tagged HDGF proteins were overexpressed and purified from HEK293T cells, and then incubated in in vitro binding systems. Anti‐HA immunoprecipitants (H) and Coomassie Blue staining (I) of the purifie HA‐tagged S6K1, myc‐tagged STAT3, and FLAG‐tagged HDGF were analyzed. (J) Primary hepatocytes were transfected with siNC or siHDGF, followed by treatment with 30 mm glucose, 100 nm insulin, and 10 nm rapamycin for 24 h.

Given that HDGF promotes STAT3 phosphorylation but lacks intrinsic kinase activity, we sought to identify the key kinases responsible for this modification. Ribosomal protein S6 kinase 1 (S6K1), a critical kinase downstream of mTOR, is activated by mTORC1 and phosphorylates downstream substrates to mediate its signaling effects [30, 31]. We hypothesized that S6K1 may mediate the HDGF‐promoted effects by regulating STAT3 phosphorylation. The interaction between HDGF and S6K1 was first verified by co‐immunoprecipitation analysis (Figure 5A). Further, pharmacological inhibition of S6K1 with LY2584702 abolished the HDGF‐promoted phosphorylation of STAT3 at Ser727 and lipogenesis in primary hepatocytes (Figure 5B), which was also confirmed in Huh7 cells (Figure S5A). Consistently, the HDGF‐S6K1 interaction was regulated by mTORC1‐S6K1 signaling, as evidenced by its reduction upon rapamycin treatment (Figure 5C) and its enhancement in TSC2‐deficient hepatocytes exhibiting hyperactivation of this pathway (Figure 5D).

Interestingly, the molecular docking analysis suggested the potential formation of a ternary complex among HDGF, S6K1, and STAT3 (Figure S5C). Co‐immunoprecipitation assays revealed that the physical interaction between S6K1 and STAT3 was strengthened by HDGF overexpression and abrogated upon HDGF deletion (Figure 5E,F). These effects were rescued by HDGF replenishment (Figure 5G), indicating that HDGF is necessary and sufficient for promoting the formation of this ternary complex. Furthermore, in vitro binding assays with purified proteins confirmed that HDGF directly facilitates S6K1‐STAT3 complex assembly (Figure 5H,I). Functionally, HDGF knockdown via siRNA in primary hepatocytes decreased the phosphorylation of STAT3 at Ser727 and lipogenic gene expression, and rapamycin further suppressed these effects (Figure 5J). These findings establish that HDGF enhances the phosphorylation of STAT3 through a mechanism dependent on S6K1.

### 3.6 Hepatocyte‐Derived Exosomal HDGF Activates Proinflammatory Macrophage Responses in MASLD

**FIGURE 6:**
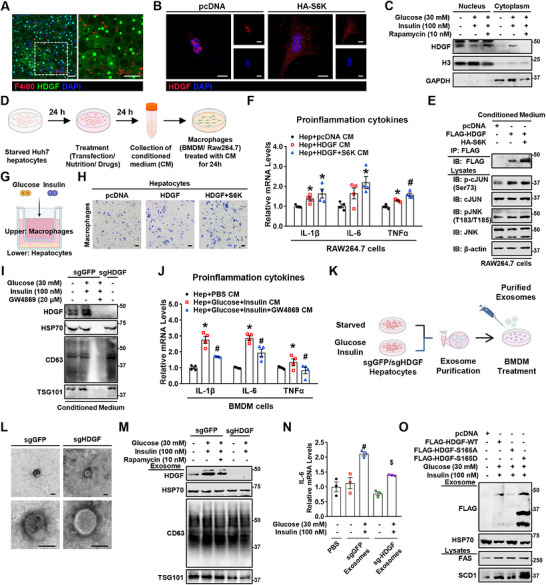
HDGF‐enriched HDGF exosome triggers proinflammatory macrophage activation. (A) Representative immunofluorescence images of HDGF in the liver of mice. Scale bar, 50 µm. (B) S6K1 promotes the nuclear‐to‐cytoplasmic translocation of HDGF. Huh7 hepatocytes were transfected with pcDNA or HA‐S6K1, and cellular localization of HDGF was analyzed by immunofluorescence staining. Scale bar, 50 µm. (C) Huh7 cells were treated with 30 mm glucose, 100 nm insulin, and 10 nm rapamycin for 24 h. Cytoplasmic and nuclear fractions were then analyzed for HDGF distribution by immunoblotting. (D–F) HDGF promotes macrophage activation via conditioned medium. RAW264.7 macrophages were incubated for 24 h with conditioned medium from Huh7 cells transfected with different plasmids and different treatments. (D) The schematic diagram of the preparation of conditioned medium and cell treatment. (E) Cell lysates of conditioned medium treated RAW264.7 macrophages were analyzed by immunoblotting. (F) mRNA levels of proinflammatory genes in conditioned medium treated RAW264.7 cells. Data were presented as mean ± SEM. n = 4. *p < 0.05 vs. pcDNA, #*p* < 0.05 vs. HDGF, one‐way ANOVA. (G,H) HDGF facilitates the chemotaxis of macrophage. Huh7 cells were transfected with pcDNA, FLAG‐HDGF, and HA‐S6K1, followed by incubated with RAW264.7 macrophages and treated with 30 mm glucose and 100 nm insulin. (G) The schematic diagram of the coculture system of RAW264.7 macrophage and Huh7 hepatocytes. (H) Migration of macrophages was assessed using crystal violet staining. Representative filters were shown. Scale bars, 50 µm. (I) Protein detection of conditioned medium derived from Huh7 hepatocytes by immunoblot. Conditioned medium from WT Huh7 cells treated with 30 mm glucose, 100 nm insulin, and 20 µm GW4869 for 24 h. (J) mRNA levels of proinflammatory genes in conditioned medium treated bone marrow derived macrophages. BMDMs were in vitro cultured and differentiated for 7 days before 24 h conditioned medium treatment. Conditioned medium from sgGFP or sgHDGF Huh7 cells treated with 30 mm glucose, 100 nm insulin, and 20 µm GW4869 for 24 h. Data were presented as mean ± SEM. n = 4. *p < 0.05 vs. PBS treated Huh7 derived conditioned medium, #*p* < 0.05 vs. glucose and insulin treated Huh7 derived conditioned medium, one‐way ANOVA. (K) The schematic diagram of the exosome purification from hepatocytes medium and followed BMDMs treatment. (L) Representative electron microscopy characterization (scale bar: 50 nm). (M) HDGF contents in purified exosomes were detected by immunoblotting. (N) HDGF sufficient exosomes trigger inflammatory macrophage activation. sgGFP or sgHDGF Huh7 cells were treated with 30 mm glucose and 100 nm insulin for exosome purification, and BMDMs were treated with derived exosomes. mRNA expression level of IL‐6 in BMDMs was detected by qPCR. Data were presented as mean ± SEM. n = 3. *p < 0.05 vs. PBS, #*p* < 0.05 vs. sgGFP‐Huh7 derived exosomes, $p < 0.05 vs. sgGFP with glucose and insulin treatment Huh7 derived exosomes, one‐way ANOVA. (O) Different HDGF mutants showed varied secretory ability by exosomes. Huh7 cells were treated with 30 mm glucose and 100 nm insulin for 12 h before exosome purification. Exosomes were analyzed by immunoblotting.

We have previously shown that hepatocyte‐derived HDGF is a potent driver of inflammation by promoting macrophage infiltration and proinflammatory gene expression during the progression of MASLD (Figure 2B,H; Figure S2B). To elucidate the precise mechanism by which HDGF modulates these inflammatory responses, we first characterized its cellular distribution. Immunofluorescence analysis revealed that HDGF was predominantly expressed in hepatocytes, with minimal expression in macrophages (Figure 6A; Figures S1I and S2A). This hepatocyte‐restricted expression pattern suggested that HDGF may function in a paracrine manner to modulate hepatic inflammation. Interestingly, S6K1 significantly promoted the translocation of HDGF from the nucleus to the cytoplasm (Figure 6B). Consistently, glucose and insulin‐induced cytoplasmic translocation of HDGF was blocked by rapamycin in Huh7 hepatocytes (Figure 6C). These data suggest that HDGF might be secreted in response to nutrient signals. Next, cell coculture assays were performed between Huh7 hepatocytes and RAW264.7 macrophages or Bone Marrow‐Derived Macrophages (BMDMs). As shown in Figure 6D–F and Figure S6A, conditioned medium (CM) from HDGF‐overexpressing hepatocytes markedly promoted the proinflammatory response of macrophages, as indicated by enhanced activation of JNK and c‐Jun, along with increased expression of proinflammatory genes, which was further enhanced by hepatocyte S6K1 overexpression. Moreover, transwell assays demonstrated that overexpression of HDGF and S6K1 enhanced macrophage migration toward hepatocytes (Figure 6G,H), suggesting that hepatocyte‐secreted HDGF may play a key role in mediating macrophage activation.

We next sought to elucidate the mechanism underlying HDGF secretion and its subsequent impact on macrophage function. Given that HDGF lacks a canonical signal peptide sequence [32], we hypothesized that it is exported via an unconventional pathway, specifically through exosome encapsulation. Initial analysis of Huh7 hepatocyte supernatants confirmed the presence of HDGF, with its level significantly upregulated following glucose and insulin stimulation (Figure 6I). Crucially, the secretion of HDGF and the subsequent proinflammatory potency of the conditioned medium toward BMDMs were markedly attenuated by the exosome biogenesis inhibitor GW4869 (Figure 6I,J).

Having established that exosome biogenesis is required for HDGF secretion, we next sought to directly validate the role of exosomal HDGF in macrophage activation. To this end, we purified exosomes from the supernatants of Huh7 hepatocytes (Figure 6K). While HDGF deficiency did not alter exosomal morphology or characteristic markers (Figure 6L; Figure S6B,C), glucose and insulin stimulation significantly enriched the HDGF cargo within these vesicles (Figure 6M). Functionally, treatment with hepatocyte‐derived exosomes triggered a robust proinflammatory response in BMDMs (Figure 6N), and this effect was largely abolished when the exosomes were harvested from HDGF‐deficient Huh7 cells (Figure 6N), establishing exosomal HDGF as a primary mediator of intercellular crosstalk. Given that Ser165 is required for the secretion of HDGF via the N‐terminus [33], the loss‐of‐function mutant HDGF(S165A) reduced the secretion levels of HDGF. Conversely, the phosphorylation mimetic mutant of Ser165 (S165D) potently induced exosomal HDGF levels (Figure 6O), suggesting that Ser165 is essential for the phosphorylation and secretory regulation of HDGF. Taken together, these findings establish that hepatic HDGF promotes macrophage activation through an exosome‐mediated secretory pathway.

### 3.7 Inhibition of STAT3 Attenuates HDGF‐Induced Hepatic Lipogenesis and MASLD

**FIGURE 7:**
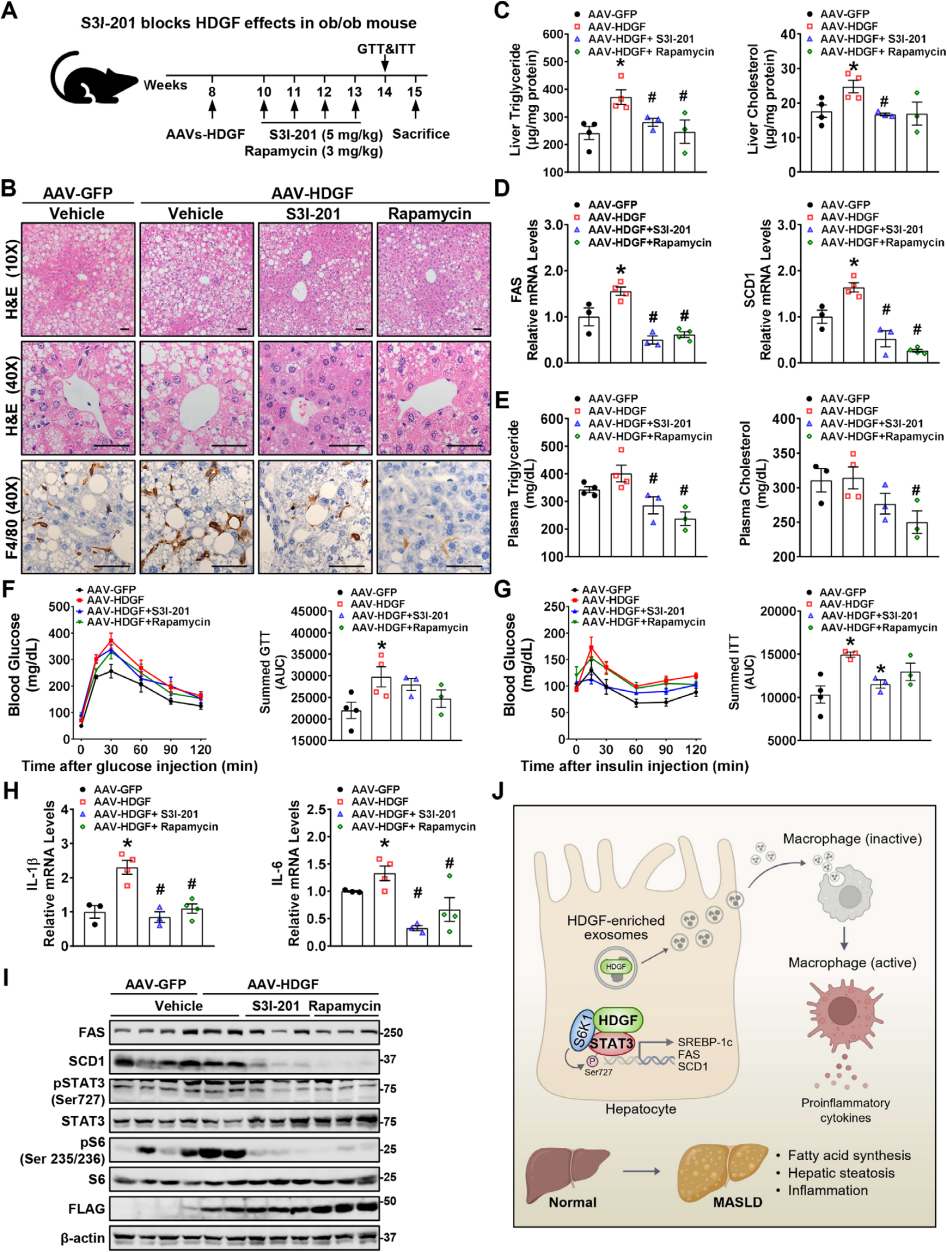
Pharmacological inhibition of STAT3 or S6K attenuates HDGF‐exacerbated MASLD. After injected with AAV‐encoding HDGF or GFP through tail vein for 2 weeks, the ob/ob mice were intraperitoneally injected with STAT3 inhibitor S3I‐201 (5 mg/ kg) or rapamycin (3 mg/ kg) 3 times per week for 4 weeks. (A) The schematic diagram of the animal experiment. (B) Representative H&E and F4/80 immunohistochemical staining in liver sections. Scale bar, 50 µm. (C) Liver triglyceride and cholesterol levels were assessed. (D) mRNA levels of lipogenic enzymes were detected by qPCR. (E) Plasma triglyceride and cholesterol levels were measured. (F) Glucose tolerance tests and respective area under the curve (AUC). (G) Insulin tolerance test and respective area under the curve (AUC). (H) mRNA levels of proinflammatory genes were detected by qPCR. Data were presented as mean ± SEM. n = 3–4. *p < 0.05 vs. AAV‐GFP, #*p* < 0.05 vs. AAV‐HDGF, one‐way ANOVA. (I) Immunoblot analysis of lipogenic genes, phosphorylation of STAT3 and S6 of mice liver lysates was performed. (J) The proposed model for hepatic HDGF as a key mediator in coordinating hepatic steatosis and intrahepatic crosstalk in MASLD. Activation of HDGF facilitates its interaction with both STAT3 and S6K1, driving the S6K1‐dependent STAT3 phosphorylation and subsequently enhancing hepatic lipogenesis. Furthermore, increased production and secretion of HDGF‐enriched exosomes trigger the intercellular macrophage proinflammatory response. Pharmacological or nanoparticle‐based neutralization approaches targeting the HDGF‐STAT3 pathway may represent a promising therapeutic strategy for MASLD.

To directly assess the therapeutic potential of targeting STAT3 and S6K1 in HDGF‐driven lipogenesis, HDGF‐overexpressing mice were treated with the STAT3 inhibitor S3I‐201 and the mTOR inhibitor rapamycin (Figure 7A). Pharmacological inhibition of STAT3 and S6K1 significantly alleviated HDGF‐induced hepatic steatosis, as evidenced by histological analysis, lipid accumulation, and expression of key lipogenic enzymes (Figure 7B–D). This intervention also improved systemic metabolic profiles, including reduced hyperlipidemia, enhanced insulin sensitivity, and improved glucose tolerance (Figure 7E–G). Importantly, inhibition of STAT3 and S6K1 attenuated the HDGF‐induced increase in liver weight and body weight (Figure S7A,B). Notably, the proinflammatory milieu was also mitigated, with a substantial decrease in F4/80^+^ macrophage infiltration and reduced expression of inflammatory cytokines (Figure 7B,H; Figure S7C,D). Consistently, protein levels of lipogenic enzymes and phosphorylation of STAT3 at Ser727 were also decreased in mice treated with S3I‐201 and rapamycin (Figure 7I). Taken together, these results demonstrate that inhibition of the S6K1‐STAT3 pathway ameliorates the hepatic lipogenesis and systemic metabolic disorders induced by HDGF overexpression.

## 4 Discussion

This study reveals HDGF as a key mediator linking hepatic steatosis and intrahepatic crosstalk between hepatocytes and macrophages during MASLD progression. Specifically, HDGF promotes lipogenesis and hepatic steatosis by facilitating S6K1‐dependent phosphorylation of STAT3 at Ser727. Moreover, HDGF is secreted from hepatocytes via an exosomal pathway, triggering a proinflammatory macrophage response, contributing to hepatic inflammation in MASLD pathogenesis (Figure 7J).

### 4.1 HDGF Acts as a Master Regulator of Hepatic Lipogenesis

A key finding of this study is the identification of HDGF as a critical regulator of STAT3‐mediated hepatic steatosis during MASLD progression. First, HDGF deficiency in hepatocytes significantly ameliorated diet‐induced hepatic steatosis and metabolic dysregulation in multiple MASLD mouse models, whereas overexpression of HDGF exacerbated hepatic and systemic metabolic dysfunction. Consistently, HDGF markedly promoted the accumulation of lipid droplets in hepatocytes. Moreover, HDGF‐driven lipogenesis and the ensuing lipotoxicity promote intrahepatic inflammatory responses, as HDGF knockout significantly attenuated macrophage infiltration and proinflammatory gene expression.

Mechanistically, this study demonstrates HDGF as an essential integrator that physically bridges S6K1 and STAT3, thereby facilitating S6K1‐dependent phosphorylation of STAT3 at Ser727 and subsequent activation of lipogenic genes. This discovery establishes a novel mechanistic link between nutrient‐sensing signaling and STAT3 phosphorylation modification in metabolic regulation, contrasting with previous characterizations of this modification primarily in the context of inflammatory signaling [34, 35, 36]. Supporting this notion, pharmacological inhibition of STAT3 by S3I‐201 effectively ameliorates hepatic steatosis and systemic metabolic disorders driven by HDGF overexpression. Collectively, our findings establish that the HDGF‐STAT3 axis plays a critical role in hepatic lipogenesis and MASLD progression.

### 4.2 Exosome‐Mediated HDGF Secretion Promotes Proinflammatory Macrophage Activation in MASLD

As a non‐classically secreted protein lacking a signal peptide, HDGF is independent of the conventional endoplasmic reticulum‐Golgi secretory pathway and is instead secreted through membrane vesicles or direct translocation under cellular stress or injury conditions [37, 38, 39]. Here, we reveal that metabolic stress induces exosomal secretion of HDGF as a key paracrine mechanism driving hepatic inflammation during MASLD pathogenesis.

Several lines of evidence support the role of hepatocyte‐derived exosomal HDGF in mediating the crosstalk between hepatocytes and macrophages. First, conditioned medium from HDGF‐overexpressing hepatocytes significantly enhanced macrophage migration and proinflammatory cytokine expression, which is consistent with the inflammatory microenvironment observed in both human MASLD liver tissues and mouse models. Notably, depletion of exosomes from the conditioned medium markedly attenuated these effects, identifying the exosomal fraction as the active mediator. Second, purified HDGF‐enriched exosomes directly triggered proinflammatory activation in macrophages, as indicated by a marked upregulation of cytokine gene levels, including IL‐1β, IL‐6, and TNFα. Importantly, this effect was significantly diminished when exosomes were derived from HDGF‐knockout hepatocytes, demonstrating that the inflammatory activity of hepatocyte‐derived exosomes is highly attributable to their HDGF cargo. Consistent with this, the metabolic stress‐induced translocation of HDGF from the nucleus to the cytoplasm provides a mechanistic basis for its role in regulating intercellular secretion. Together, these findings establish a pivotal role for hepatocyte‐driven exosomal HDGF in regulating proinflammatory macrophage activation and MASLD progression.

### 4.3 Therapeutic Potential of Targeting HDGF in MASLD

The current finding identifies HDGF as a promising therapeutic target for MASLD, supported by clinical associations and mechanistic insights. The clinical relevance of our findings is evidenced by elevated HDGF expression in the liver and circulation of patients with MASLD, along with a positive correlation between circulating HDGF levels and key clinical parameters, including BMI, triglyceride, and cholesterol levels. Furthermore, hepatocyte‐specific genetic deletion of HDGF resulted in marked attenuation of both hepatic steatosis and inflammation, underscoring its critical role in MASLD pathogenesis. Collectively, these findings indicate that overactivation of HDGF contributes to hepatic metabolic dysregulation, whereas inhibition of HDGF may confer potential therapeutic benefit in MASLD.

From a therapeutic perspective, the HDGF‐S6K1‐STAT3 axis presents multiple potential nodes for pharmacological intervention. First, S6K1 inhibition significantly decreases the phosphorylation of STAT3 and represents a novel therapeutic strategy with greater precision. Moreover, disruption of STAT3 phosphorylation at Ser727 selectively suppresses lipogenic signaling, whereas Tyr705 phosphorylation is dispensable for this process, which may provide a strategic basis for designing site‐specific therapeutic interventions. Targeting this axis clinically may be achieved through liver‐specific strategies, including nanoparticle‐based HDGF neutralization, modulation of exosome secretion, or direct disruption of the HDGF‐STAT3 complex with small molecules. Given the pleiotropic roles of HDGF in physiological processes, such targeted approaches could simultaneously ameliorate both metabolic and inflammatory components of MASLD while minimizing potential systemic effects.

Despite establishing hepatocyte‐derived HDGF as a critical driver of MASLD progression, several limitations warrant acknowledgment. First, while our genetic models establish a clear causal role for HDGF, the therapeutic efficacy of direct pharmacological intervention remains to be explored. Second, the role of HDGF in more advanced stages of MASLD, particularly in the progression to MASH, requires further investigation. Moreover, although we successfully rescued the phenotype by targeting the downstream effectors S6K1 and STAT3, direct inhibition of HDGF via neutralizing antibodies, small molecule inhibitors, or antisense oligonucleotides may represent a more upstream and potentially more comprehensive therapeutic strategy with reduced off‐target effects. Addressing these questions will require rigorous preclinical validation in diverse animal models and subsequent clinical trials to definitively assess the safety and efficacy of HDGF‐directed therapies in ameliorating MASLD and its associated metabolic disorders.

In summary, this study identifies HDGF as a key regulator that integrates metabolic and inflammatory signals in MASLD pathogenesis in both mice and humans. HDGF promotes hepatic lipogenesis by facilitating S6K1‐dependent STAT3 phosphorylation. In parallel, hepatocyte‐derived exosomal HDGF drives macrophage proinflammatory activation, thereby coupling hepatic steatosis with inflammation during disease progression. Collectively, these findings establish that targeting the HDGF‐STAT3 axis, via pharmacological or nanoparticle‐mediated approaches, may represent a promising therapeutic strategy for simultaneously ameliorating both metabolic dysfunction and hepatic inflammation in MASLD.

## Author Contributions

J.W., D.D., A.C., Y.L., W.S., C.G., and W.F. contributed to experiment design. J.W., D.D., Z.Z., X.C., W.L., J.S., Z.H., P.T., J.B., X.F., B.X., P.H., L.X., H.Y., Y.W., H.L., and W.S. contributed to the acquisition and analysis of data. Z.J., Y.Long, T.Z., M.Y., Y.J., Y.X., and C.G. provided reagents and material support. A.C., H.L., Q.D., Y.X., F.S., and Y.L. edited the manuscript with important intellectual content. J.W., A.C., W.S., F.S., and W.F. obtained the funding. J.W., D.D., Y.L., W.S., C.G., and W.F. wrote the manuscript.

## Funding

This work was supported by grants from Noncommunicable Chronic Diseases‐National Science and Technology Major Project (2024ZD0531300) and National Natural Science Foundation of China (82302419) to J.W. This work was supported by the grants from National Natural Science Foundation of China (82570678, 82170587) and Sichuan Provincial Natural Science Foundation for Outstanding Youth Foundation (2024NSFJQ0054) to W.F. This work was also supported by the Construction Project of the Discipline Peak‐Climbing Plan of Xinhua Hospital Affiliated to Shanghai Jiao Tong University School of Medicine (XKPF2024B404) to F.S. This work was supported by grants from National Natural Science Foundation of China (32371206), Shanghai Rising‐Star Program (24QA2711400) and Open Project Program of Metabolic Vascular Diseases Key Laboratory of Sichuan Province (2023MVDKL‐K2) to A.C. This work was supported by grants from Postdoctoral Fellowship Program of CPSF (GZC20251002), Natural Science Foundation of Shanghai (24ZR1478200) and Open Project Program of Metabolic Vascular Diseases Key Laboratory of Sichuan Province (2025MVDKL‐K3) to W.S.

## Conflicts of Interest

The authors disclose no conflicts of interest.

## Supporting information




**Supporting File 1**: advs75467‐sup‐0001‐SuppMat.docx.

**Supporting File 2**: advs75467‐sup‐0002‐FigureS1‐S7.zip.

**Supporting File 3**: advs75467‐sup‐0003‐TableS1.docx.

## Data Availability

The data that support the findings of this study are available from the corresponding author upon reasonable request.
