# Supplementary material for: Hepatoma‐Derived Growth Factor Coordinates STAT3 Pathway and Exosome‐Mediated Intrahepatic Crosstalk to Control Hepatic Steatosis and MASLD
Source: Adv Sci (Weinh). 2026 May 1;13(42):e23964. doi: 10.1002/advs.202523964 (PMC13335579; doi:10.1002/advs.202523964)
Supplement: Supplementary file 1 — Supporting File 1: advs75467‐sup‐0001‐SuppMat.docx. [file ADVS-13-e23964-s001.docx]

**Hepatoma-Derived Growth Factor Coordinates STAT3 Pathway and Exosome-Mediated Intrahepatic Crosstalk to Control Hepatic Steatosis and MASLD**

Jian Wen^1,2,3,^^9^, Dong Ding^3,9^, Zengpeng Zheng^3^, Xufeng Chen^3^, Wenjing Li^3^, Jiaxin Shen^3^, Zhiwei Huang^1,2^, Peng Tan^1,2^, Junjie Bai^1,2^, Xia Fang^4^, Baofang Xing^3^, Puyuan Hu^3^, Linghao Xu^4^, Haokai Yu^3,5^, Yixi Wang^3^, Zongzhe Jiang^4^, Yang Long^6^, Tiejun Zhou^7^, Mingxin Ye^1,2^, Yu Jiang^1,2^, Aoyuan Cui^3^, Hong Li^3^, Qiurong Ding^3^, Yong Xu^4^, Yu Li^3^**,** Weitong Su^3^*****, Feng Shen^8^***,** Chenlin Gao^4^*****, Wenguang Fu^1,2^*****

^1^Department of General Surgery (Hepatobiliary Surgery), Biliary-Pancreatic Center, The Affiliated Hospital, Southwest Medical University, Luzhou, Sichuan 646000, China; ^2^Metabolic Hepatobiliary and Pancreatic Diseases Key Laboratory of Luzhou City, Academician (Expert) Workstation of Sichuan Province, The Affiliated Hospital, Southwest Medical University, Luzhou, Sichuan 646000, China; ^3^Shanghai Institute of Nutrition and Health, University of Chinese Academy of Sciences, Chinese Academy of Sciences, Shanghai 200031, China; ^4^Department of Endocrinology and Metabolism, Metabolic Vascular Disease Key Laboratory of Sichuan Province, The Affiliated Hospital of Southwest Medical University, Luzhou, Sichuan 646000, China; ^5^State Key Laboratory of Food Nutrition and Safety, College of Biotechnology, Tianjin University of Science and Technology, Tianjin 300457, China; ^6^Experimental Medicine Center, Department of Endocrinology and Metabolism, Metabolic Vascular Disease Key Laboratory of Sichuan Province, The Affiliated Hospital of Southwest Medical University, Luzhou, Sichuan 646000, China; ^7^Department of Pathology, The Affiliated Hospital, Southwest Medical University, Luzhou, Sichuan 646000, China; ^8^Department of Gastroenterology & Endoscopy, Xinhua Hospital Affiliated to Shanghai Jiao Tong University School of Medicine, Shanghai 200092, China; ^9^These authors contribute equally to this work.

**Running Title:** **Intrahepatic HDGF signaling regulates hepatic steatosis**

**Supplemental Figure Legends**

**Fig.S1. HDGF is positively correlated with lipid synthesis and inflammation.** (A) RNA-seq analysis of differentially expressed genes between human MASLD and control samples in GSE126848 database. Heatmap showing the global transcriptional profile of differentially expressed genes, including the up-regulation of HDGF in MASLD. (B) KEGG enrichment analysis showed the significantly enriched biological process. (C) Single-cell analysis revealed hepatocytes as the major cellular source of HDGF in the liver. (D-E) Correlation of plasma HDGF levels with triglyceride (D) and cholesterol levels (E) in humans. n = 12. Pearson correlation analysis. (F-G) Increased mRNA levels of lipogenic genes (F) and proinflammatory genes (G) in the MASLD livers of humans. Data were presented as mean ± SEM. n = 3-6, one-way ANOVA. (H) Quantification of positive signals in HDGF immunohistochemical staining, Oil red O staining, and CD68 immunohistochemical staining in the liver sections of control and MASLD humans. Data were presented as mean ± SEM. n = 3, unpaired two-tailed Student's t-test. (I) Representative histology of human and mice livers. Panels show immunofluorescence staining for hepatocytes (HNF4α) and myeloid cells (CD68 for human, F4/80 for mice). Scale bar, 50 μm.

**Fig.S2. Hepatocyte-specific knockout of HDGF by AAV-mediated CRISPR-Cas9 improves hepatic steatosis.** (A) Representative histology of AAV-sgRNA injected mice livers. Panels show immunofluorescence staining for hepatocytes (HNF4α), macrophages (F4/80) and hepatic stellate cells (Desmin). Scale bar, 50 μm. (B) Quantification of positive signals in oil red O and F4/80 immunohistochemical staining in the liver sections of sgGFP control mice and sgHDGF mice. Data were presented as mean ± SEM. n= 3. *p < 0.05, unpaired two-tailed Student's t-test.

**Fig.S3. Hepatocyte-specific overexpression of HDGF exacerbates hepatic steatosis and systemic glucose intolerance in ob/ob mice.** (A) Representative H&E staining in the liver sections of mice. Scale bar, 50 μm. (B) Liver lysates were analyzed by immunoblots. (C) HDGF overexpression increases liver cholesterol and liver triglyceride levels. (D) HDGF overexpression aggravates plasma triglyceride and cholesterol levels. (E) Increased plasma ALT and AST levels were observed in hepatocyte-specific HDGF overexpression mice. (F) HDGF overexpression aggravates glucose tolerance in mice. Glucose tolerance tests and respective area under the curve (AUC) were shown. Data were presented as mean ± SEM. n = 4-10. *p < 0.05, unpaired two-tailed Student's t-test. (G) HDGF promotes glucose and insulin stimulated lipid accumulation in primary hepatocytes. Representative BODIPY staining was performed. Scale bar, 50 μm. (H) Triglyceride content was measured in primary hepatocytes. Primary hepatocytes were transfected with overexpression plasmids, then treated with 30 mM glucose and 100 nM insulin for 24 hours. Triglyceride content was measured and adjusted by protein content. Data were presented as mean ± SEM. n = 6. *p < 0.05 vs. siNC and PBS, #p < 0.05 vs. siHDGF and PBS, one-way ANOVA.

**Fig.S4. HDGF promotes STAT3 transcriptional activation and facilitates its physical interaction with S6K1.** STAT3 ChIP-seq profiling of sgGFP and sgHDGF huh7 cells under starvation or glucose and insulin treatment for 24 hours. GO enrichment analysis (A) and KEGG enrichment analysis (B) were shown. (C-E) Distribution of chromatin regions or peaks in genomic regions of the ChIP-seq analysis in sgGFP vehicle (C), sgGFP glucose and insulin (D), sgHDGF glucose and insulin (E). (F) The luciferase activities of STAT3-Luc in HEK293T cells. HEK293T cells were transfected with STAT3-responsive p2×SIE-Luc reporter plasmid and Renilla luciferase reporter plasmid pRL-SV40, together with pcDNA as indicated, and then treated with 10 ng/ml IL-6. Subsequently, cells were treated with 50 nM pan-STAT3 inhibitor S3I-201 or 30 μM pSTAT3 (Tyr705)-specific inhibitor cryptotanshinone for an additional 12 h. Dual luciferase activities were measured. Data were presented as mean ± SEM. n = 6. *p < 0.05 vs. pcDNA and vehicle, #p < 0.05 vs. pcDNA with IL-6, one-way ANOVA.

**Fig.S5. Physical interaction between HDGF, STAT3 and S6K1.** (A) Huh7 cells were transfected with FLAG-HDGF plasmid and treated with 30 mM glucose and 100 nM insulin for 12 h, then with subsequent treated with 200 nM S6K1 inhibitor LY2584702 for another 12 h. Lysates were analyzed by immunoblots. (B) sgRNA control (sgGFP) and HDGF deficient (sgHDGF) Huh7 cells were treated with 30 mM glucose, 100 nM insulin and 10 nM rapamycin for 24 h. Lysates were analyzed by immunoblots. (C) Molecular docking analysis revealed that HDGF forms a stable ternary complex with S6K1 and STAT3, creating complementary binding interfaces among these proteins. The protein-protein docking was performed in an aerial view and lateral view by Alphafold3 and visualized by PyMOL.

**Fig.S6. Exosomes derived from HDGF-deficient Huh7 cells reduced proinflammatory cytokine genes expression.** (A) Transfected efficiency of Huh7 cells was detected by immunoblotting. (B-C) HDGF deficiency had no significant effect on exosome size. Exosomes derived from sgGFP (B) and sgHDGF (C) Huh7 cells exhibited comparable sizes, detected by Nanoparticle Tracking Analysis (NTA). (D) HDGF sufficient exosomes trigger inflammatory macrophage activation. sgGFP or sgHDGF Huh7 cells were treated with 30 mM glucose and 100 nM insulin for exosome purification, and BMDM macrophages were treated with derived exosomes. mRNA expression levels of IL-1β and TNFα in BMDMs were detected by qPCR. Data were presented as mean ± SEM. n = 3. *p < 0.05 vs. PBS, #p < 0.05 vs. sgGFP-Huh7 derived exosomes, $ p < 0.05 vs. sgGFP with glucose and insulin treatment Huh7 derived exosomes, one-way ANOVA.

**Fig.S7. Pharmacological inhibition of STAT3 or S6K attenuates HDGF-exacerbated MASLD.** After being injected with AAV-encoding HDGF or GFP through tail vein for 2 weeks, the ob/ob mice were intraperitoneally injected with STAT3 inhibitor S3I-201 (5 mg/ kg) or rapamycin (3 mg/ kg) 3 times per week for 4 weeks. (A) The ratio of liver weight to body weight. (B) The ratio of gain weight to body weight. (C) mRNA level of TNFα was detected by qPCR. (D) Quantification of positive signals in F4/80 immunohistochemical staining in the liver sections. Data were presented as mean ± SEM. n= 3-4. *p < 0.05 vs. AAV-GFP, #p < 0.05 vs. AAV-HDGF, one-way ANOVA.

**Supplemental Experimental Procedures**

| **Reagents** | **Company** | **Catalog number** |
| --- | --- | --- |
| D-(+)-Glucose | Sigma | G8270-100G |
| Insulin (human) recombinant | TOCRIS Bioscience | 3435 |
| Recombinant Mouse IL-6 | Novoprotein | CG39 |
| Recombinant Mouse/Rat TGF-beta 1 | Novoprotein | CK33 |
| Collagenase | Sigma | C2139-100MG |
| Collagenase, Type 1 (CLS-1),1 gm | worthington | LS004197 |
| Deoxyribonuclease I from bovine pancreas (DNase I) | Sigma | DN25-100MG |
| Palmitic acid | Sigma | P0500-10G |
| TRIzol Reagent | Life Technologies | 15596026CN |
| Mouse HDGF ELISA Kit | LDQB | LDQB-29886 |
| Human Hepatoma-derived growth factor (HDGF) ELISA kit | ABclonal | RK11234 |
| VEX Exosome Isolation Reagent | Vazyme | R601 |
| HiScript III RT SuperMix for qPCR (+gDNA wiper) | Vazyme | R323-01 |
| ChamQ SYBR qPCR Master Mix (High ROX Premixed) | Vazyme | Q341-02 |
| TruePrep DNA Library Prep Kit V2 for Illumina | vazyme | TD501 |
| TruePrep Index Kit V2 for Illumina | vazyme | TD202 |
| VAHTS DNA Clean Beads | vazyme | N411-01 |
| VAHTS DNA Adapters set1 for Illumina | vazyme | N801-01 |
| HiScript III RT SuperMix for qPCR (+gDNA wiper) | vazyme | R323-01 |
| ChamQ SYBR qPCR Master Mix (High ROX Premixed) | vazyme | Q341-03 |
| NSC 74859 (S3I-201) | Selleck | S1155 |
| Cryptotanshinone | MedChemExpress | HY-N0174 |
| Rapamycin | Sigma-Aldrich | 553210 |
| LY2584702 | Selleck | S7698 |
| GW4869 | MedChemExpress | HY-19363 |

| **Antibody (Clone number)** | **Company** | **Catalog number** | |
| --- | --- | --- | --- |
| Anti-HDGF Antibody | Abcam | | Ab128921 |
| S6K1 Antibody | Cell Signaling Technology | | 9202 |
| Phospho-Thr389 S6K1 Antibody | Cell Signaling Technology | | 9234 |
| Phospho-Stat3 (Ser727) Antibody | Cell Signaling Technology | | 9134 |
| Phospho-Stat3 (Tyr705) Antibody | Cell Signaling Technology | | 9131 |
| Stat3 (124H6) Mouse Antibody | Cell Signaling Technology | | 9139 |
| Stat3 (D3Z2G) Rabbit Antibody | Cell Signaling Technology | | 12640 |
| mTOR Antibody | Cell Signaling Technology | | 2972 |
| Phospho-mTOR (Ser2448) Antibody | Cell Signaling Technology | | 2971 |
| Fatty Acid Synthase (C20G5) Rabbit Antibody | Cell Signaling Technology | | 3180 |
| SCD1 (C12H5) Rabbit Antibody | Cell Signaling Technology | | 2794 |
| S6 Ribosomal Protein (5G10) Rabbit Antibody | Cell Signaling Technology | | 2217 |
| Phospho-S6 Ribosomal Protein (Ser235/236) Rabbit Antibody | Cell Signaling Technology | | 4858 |
| IRS-1 Antibody | Cell Signaling Technology | | 2382 |
| Phospho-IRS-1 (Ser307) Antibody | Cell Signaling Technology | | 2381 |
| Anti-β-actin Antibody | Santa Cruz | | sc-69879 |
| HDGF Rabbit Antibody | ABclonal | | A0589 |
| myc-Tag Rabbit Antibody | ABclonal | | AE070 |
| HA-Tag Rabbit Antibody | ABclonal | | AE105 |
| DDDDK-Tag Rabbit Antibody | ABclonal | | AE092 |
| HSP70 Rabbit Antibody | ABclonal | | A23457 |
| GAPDH Rabbit Antibody | ABclonal | | A19056 |
| TSC2 Rabbit Antibody | ABclonal | | A19540 |
| Pan-Phospho-Serine/Threonine Mouse Antibody | ABclonal | | AP1067 |
| CD63 Polyclonal antibody | Proteintech | | 25682-1-AP |
| TSG101 Polyclonal antibody | Proteintech | | 28283-1-AP |

**Human liver specimens.** Liver samples were obtained from adult patients undergoing resection of benign liver disease such as liver hemangioma, liver trauma or gallstone disease. MASLD and nondiabetic control liver specimens were acquired from these patients diagnosed with or without MASLD. Part of the liver specimens was preserved in formalin, and the other part of the same liver biopsy was placed on dry ice within a few minutes after removal from the liver and then stored in liquid nitrogen before use for molecular biology analysis.

**Animal model and diet.** All mouse lines were maintained on a C57/BL6 background. 8-week-old male mice and ob/ob mice were purchased from Gempharmatech Co. Ltd, China. Mice were fed on the standard chow diet or a high-fat high-sucrose (HFHS) diet. All mice were housed under a 12:12-h light/dark cycle at controlled temperature. As for AAV-mediated HDGF liver specific knockout mice, to achieve hepatocyte-specific gene ablation, we engineered an AAV vector with dual functionality: sgRNA delivery and Cre recombinase expression driven by the hepatocyte-specific TBG promoter. As Cas9 expression in these mice is Cre-dependent, genome editing is initiated exclusively in Cre-expressing hepatocytes. This design ensures precise and restricted gene knockout within the hepatocytes. All animal experimental protocols were approved by the Institutional Animal Care and Use Committee at Shanghai Institute of Nutrition and Health, Chinese Academy of Sciences.

**Protein sample preparation for mass spectrum.** Cell lysates prepared from Huh7 cells stably expressing FLAG-tagged HDGF were incubated with anti-FLAG-conjugated beads. The immunoprecipitated proteins were resolved by SDS-PAGE and stained with Coomassie Brilliant Blue dyes. Protein bands of FLAG-HDGF were excised and digested with trypsin buffer at 37°C overnight. The resultant peptides were subjected to NSI source followed by tandem mass spectrometry (MS/MS) in Q ExactiveTM Plus (Thermo) coupled online to the UPLC. To generate an extracted ion chromatogram, the raw data were processed and analyzed using Proteome Discoverer 1.3 software (PTM Biolabs, China).

**Metabolic phenotyping.** For glucose tolerance tests (GTT), mice were fasted for 16 h followed by intraperitoneal injection with glucose solution (1 g/kg body weight). For insulin tolerance tests (ITT), mice were fasted for 6 h, and intraperitoneally injected with 1 U/kg insulin solution. Blood glucose was determined at 0, 15, 30, 60, 90, and 120 min after injection of glucose or insulin. Triglycerides determination kit (Thermo Fisher Scientific), cholesterol determination kit (Thermo Fisher Scientific) and insulin ELISA kit (Millipore) was used for plasma, respectively according to the manufacturer’s instructions. For body composition analysis, whole-body composition of live mice was analyzed using NMR technology (EchoMRI, Houston, TX, USA). Mouse body weight was monitored weekly.

**Liver histological analysis.** The livers were fixed in 10% phosphate-buffered formalin acetate at 4°C overnight and then embedded in paraffin wax. Paraffin sections (5 μm) were cut and mounted on glass slides for hematoxylin and eosin (H&E) staining, as well as Sirius Red staining according to standard protocols.

**Cell culture and treatment.** The human Huh7 and HEK293T cells were purchased from Cell Bank, Type Culture Collection Committee, Chinese Academy of Sciences (Shanghai, China). The Huh7 and HEK293T cells were cultured in DMEM containing 4.5 g/L glucose,10% fetal bovine serum (FBS) and 1% penicillin–streptomycin (P/S) as described previously[^1^](#_ENREF_1), and cells were incubated in a humidified atmosphere of 5% CO_2_ at 37 °C and passaged every 2 days by trypsinization. Cells were transfected with plasmids for 24 hours, followed by treatment with insulin and glucose for 24 hours respectively.

**Mouse primary hepatocytes isolation and culture.** Primary mouse hepatocytes were isolated using a method described previously[^1^](#_ENREF_1)^,^ [^2^](#_ENREF_2). Briefly, mice were anesthetized with sodium pentobarbital (30 mg/kg intraperitoneally), and the portal vein was cannulated under aseptic conditions. The liver was perfused with ethylene glycol-bis (2-aminoethylether)-N,N,N',N'-tetraacetic acid (EGTA) solution (5.4 mmol/l KC1, 0.44 mmol/l KH2PO4, 140 mmol/l NaCI, 0.34 mmol/l Na2HPO4, 0.5 mmol/l EGTA, 25 mmol/l Tricine, pH 7.2) and Hank’s Balanced Salt Solution (HBSS) containing 0.075% collagenase type I (Sigma-Aldrich), 10 mg/ml DNase I (Sigma-Aldrich), 200 units/ml penicillin, and 200 μg/ml streptomycin, and then digested with 0.025% collagenase solution for the mouse liver. The isolated mouse hepatocytes were then cultured at 80%-90% confluence in DMEM medium containing 10% FBS in rat-tail collagen type I-coated 6-well plates (BD Biosciences) overnight.

**Mouse BMDMs isolation.** BMDMs were isolated from 6- to 8-week-old C57BL/6J mice[^3^](#_ENREF_3). Briefly, femurs and tibias were dissected, sterilized in 70% ethanol, and bone marrow was flushed with RPMI 1640 using a 25G needle. The cell suspension was centrifuged (1300 rpm, 10 min), and erythrocytes were lysed with RBC lysis buffer (3 min). After neutralization with PBS, cells were plated in complete DMEM supplemented with 20 ng/mL recombinant M-CSF at a density of 1×10⁶ cells/mL in non-tissue culture-treated dishes and incubated at 37°C with 5% CO₂ for 7 days. Fresh medium was added on day 3. Differentiated BMDMs were harvested on day 7 by gentle scraping or PBS/EDTA treatment, followed by centrifugation (300 × g, 5 min) for subsequent experiments.

**Exosome Isolation.** Exosomes were isolated from cell culture supernatant by differential centrifugation. Briefly, conditioned medium from cells cultured in serum-containing medium was centrifuged at 300 × g for 10 min to remove cells, followed by filtration through a 0.22 μm syringe filter. The filtered supernatant was ultracentrifuged at 10,000 × g for 30 min at 4°C to pellet exosomes. After careful removal of the supernatant, the pellet was washed with PBS and centrifuged again at 1,500 × g for 2 min. Finally, the exosome pellet was resuspended in sterile PBS for downstream analysis.

**Plasmids.** The plasmid encoding FLAG-tagged HDGF, HA-tagged S6K1, myc-tagged STAT3 were constructed by cloning complementary DNA (cDNA) as indicated into the sites of pcDNA3.1 or pCDH vectors (pCDH-CMV-FLAG-HDGF). To generate the HDGF overexpression plasmid, the cDNA encoding GFP in AAV-TBG-GFP vector was replaced with a fragment encoding cDNA sequence of HDGF in the adeno-associated virus shuttle plasmids (pAAV-TBG-HDGF). For the knockdown construct, the AAV-shHDGF plasmid was produced by substituting the GFP coding sequence in the AAV-GFP-U6-MCS vector with a short hairpin RNA (shRNA) sequence targeting HDGF (pAAV-TBG-shHDGF). For CRISPR/Cas9-mediated knockout, single-guide RNA (sgRNA) sequences targeting HDGF were ligated into a pAAV-U6-sgRNA vector (pAAV-TBG-sgHDGF). Plasmids were transferred by PEI or lipofectamine 2000, and the medium was changed after 8 h. Cells were treated indicated after 24-36 hours and analyzed.

**Generation of gene knockout cell lines by CRISPR-Cas9.** To generate HDGF knockout cell lines, guide RNAs were cloned into LentiCrispr v2 (Addgene, MA) as described previously[^4^](#_ENREF_4). Huh7 cells were transfected with plasmids targeting specific genes, and then selected with puromycin for 48 hours. The survived cells were trypsinized and plated in 96-well plates with approximately one cell per well. The single-cell clones would expand in two weeks, and the knockout cells were verified by immunoblotting for targeted proteins and DNA sequencing. The genomic primers for sgRNA cloning were designed using the Zhang laboratory CRISPR design tool (<http://chopchop.cbu.uib.no/>), and the sequences involved here were listed below: 1) sgGFP: GGGCGAGGAGCTGTTCACCG; 2) mouse sgHDGF: AGAGTACAAGTGCGGAGACC; 3) human sgHDGF: CCGCCATGTCGCGATCCAAC.

**Small interfering RNA (siRNA) knockdown.** Knockdown experiments of HDGF in Huh7 cells were performed using siRNA oligonucleotides from Sangon Biotech (Shanghai, China). The sequences of the siRNA oligos are as follows: siHDGF, ACUACAACUCCCAAAACUCUU (sense), GAGUUUUGGGAGUUGUAGUUA (anti-sense); Negative control siRNA (siNC), UUCUCCGAACGUGUCACGU. Cells were transfected with siRNAs using Hieff Trans TM in virto siRNA/miRNA transfection regent (cat. 40806ES, YEASEN).

**short-hairpin RNA (shRNA) knockdown.** The short hairpin RNAs targeting mouse HDGF and negative control were purchased from Sangon Biotech (Shanghai, China). The following shRNA targeting sequences were used: sh-HDGF: GCCAACAAATACCAAGTCTTT; shGFP, TTCTCCGAACGTGTCACGT.

**Lentivirus package, infection and selection**. Lentiviral particles were generated as described previously[^1^](#_ENREF_1). Briefly, HEK293T cells were transfected with lentivirus shuttle plasmids pCDH-CMV-FLAG-HDGF and packing plasmids pMDLg/pRRE, pRSV-Rev and pMD2.G in a molar ratio of 3:1:1:1. The supernatant of lentivirus particles were collected after 48 hours transfection and centrifuged at 50000 x g for 2 hours at 4°C. For lentivirus infection, HEK293T cells were infected with lentiviruses for 24 hours and then passaged and selected in media containing puromycin for generation of cell lines stably expressing HDGF.

**Generation of adeno-associated viruses (AAV) and *in vivo* delivery.** Adeno-associated viruses were generated as described previously. Adeno-associated virus serotype 8 (AAV8) under the control of the thyroid binding globulin promoter (TBG) that only infects hepatocytes was used to target hepatocytes in mouse livers. Briefly, HEK293T cells were transfected with AAV shuttle vector plasmids, Delta F6 (DF6) helper plasmid that supplies E2a, E4, VARNA and AAV2/8 RC plasmid. The ratio of the above three vectors is 1:1:1. Following transient transfection with PEI and virus package in HEK293T cells, AAV vectors were purified using discontinuous iodixanol gradient centrifugation as previously described with minor modifications. The purified AAV vectors were dissolved and stored in 1xPBS containing 10^-4^ F188 and 5% glycerol, and tittered using a previously described real-time PCR procedure. Adeno-associated viruses (approximately 1.0 × 10^11^ viral particles per mouse) were delivered into mice by tail vein injection.

**Nuclear and cytoplasmic extraction.** Nuclear and cytosolic extraction was performed using the Nuclear and Cytoplasmic Protein Extraction Kit (cat. P0028) obtained from Beyotime (Shanghai, China). Briefly, Huh7 cells and HEK293T cells were treated by forskolin for 2 hours, harvested with trypsin–EDTA and then centrifuged at 500×g for 5 minutes. Next, the cell pellets were washed with PBS, and centrifuged at 500 × g for 5 minutes. To get the cytoplasmic extracts, the supernatant was removed and discarded, 200 μl ice-cold cytoplasmic protein extraction reagent was added to the cell pellet. The tube was vortexed vigorously for 5 seconds, then the tube was incubated on ice for 10 minutes. 11 μl ice-cold cytoplasmic protein extraction reagent was added to the tube, followed by vortex and centrifugation at 16,000 g for 5 minutes. The supernatant containing the cytoplasmic fraction was transferred to a new tube. To get the nuclear extract, the pellet fraction was suspended in 50 μl ice-cold nuclear protein extraction reagent, the tube was vortexed vigorously for 15 seconds, and the sample was placed on ice and vortexed for 15 seconds every 2 minutes for a total of 30 minutes. After centrifuge at 16,000 g for 5 minutes, the supernatant containing the nuclear fraction was transferred to a new tube. The cytoplasmic and nuclear extracts were collected and used for immunoblots.

**Immunofluorescence** **staining.** For immunofluorescence live cell staining, cells were attached to glass coverslips coated with poly-d-lysine (for Huh7 cells and primary hepatocytes) or not (for HEK293T cells) in appropriate medium. Cells were washed with ice cold PBS and fixed with 4% paraformaldehyde (PFA), then the cells were permeabilized with 1% Triton X-100 for 5 min and blocked with 1% BSA (Sigma) for 30min. Next, cells were incubated in primary antibody (1:200 dilution) at 4°C overnight, followed by incubation with secondary antibody (1:400 dilution) at 37°C for 60 min and DAPI staining for 10 min. For immunofluorescence staining of liver tissue paraffin sections, the samples were cut into 2-5 μm slices and dewaxed, rehydrated and permeated. After being restored to room temperature, the sections of liver were soaked in PBS for 10min and then blocked by 1% BSA for 30min. Next, cells were incubated in primary antibody (1:200 dilution) at 4°C overnight, followed by incubation with secondary antibody (1:400 dilution) at 37°C for 60 min and DAPI staining for 10 min. Coverslips were mounted, sealed with nail polish and assessed with fluorescence microscopy. Images were acquired through 63x oil objective lens with a Zeiss LSM880 confocal laser scanning microscope.

**Immunoblots and immunoprecipitation analysis.** Immunoblotting analysis was carried out as described previously[^5^](#_ENREF_5)^,^ [^6^](#_ENREF_6). Tissues and cells were lysed in NP-40 lysis buffer (50mM Tris-HCl pH 8.0, 150mM NaCl, 1% NP-40, 5 mM EDTA, 1 mM EGTA, 1 mM sodium orthovanadate, 10 mM sodium fluoride, 1 mM phenylmethylsulfonyl fluoride, 2 μg/ml aprotinin, 5 μg/ml leupeptin, and 1 μg/ml pepstatin). Lysates were centrifuged, and the supernatant was detected protein concentration by BCA. For immunoblotting, total protein (20-50 μg per lane) was separated by 10% sodium dodecyl sulfate-polyacrylamide gel electrophoresis (SDS–PAGE) and transferred to polyvinylidene difluoride (PVDF) membranes in a transfer buffer consisting of 25 mM Tris base, 190 mM glycine, and 20% methanol. The membranes were blocked by 5% non-fat milk in Tris-buffered saline with 0.1% Tween 20 (TBST) and incubated with primary antibody followed by horseradish peroxidase-conjugated secondary antibody. For immunoprecipitation, cell lysates containing overexpressed recombinant or endogenous proteins were incubated with specific primary antibodies overnight, and protein A/G Sepharose beads were added for another 4 hours at 4ºC. The precipitates were washed three times with ice-cold lysis buffer and analyzed by immunoblots.

***In vitro* protein purification.** FLAG-HDGF, HA-S6K1, and myc-STAT3 were purified from transfected Huh7 Cells or HEK293T cells lysed in NP-40 buffer (20 mM Tris-HCl pH 7.5, 150 mM NaCl, 1% NP-40, protease inhibitors). FLAG-HDGF was isolated by anti-FLAG M2 affinity resin with 3×FLAG peptide elution, HA-S6K1 was purified using anti-HA agarose beads eluted with HA peptide, and myc-STAT3 was captured by anti-myc magnetic beads with myc peptide elution. All proteins were dialyzed into storage buffer (20 mM HEPES pH 7.4, 150 mM NaCl, 10% glycerol), concentrated using Amicon Ultra devices, and verified by SDS-PAGE and western blotting with respective tag antibodies.

**Dual luciferase activity assays.** HEK293T cells were co-transfected with 0.5 μg of empty vector or plasmids encoding HDGF as indicated, along with 0.5 μg STAT3-responsive luciferase reporter (p2×SIE-Luc) and 80 ng of Renilla luciferase plasmid pRL-SV40 (Promega) as an internal control in 12 well plates. 24 hours post transfection, the media was changed, and the cells were allowed to recover for additional 18 hours. Dual luciferase assays for Firefly luciferase and Renilla luciferase activities were performed in duplicates according to the manufacturer’s protocols (Promega). Luciferase activity was measured using an Infinite 2000pro plate reader (TECAN, Switzerland). The Firefly luciferase activity was normalized to the Renilla luciferase activity (Firefly luciferase/Renilla luciferase) and presented as relative luciferase activity.

**ChIP sequencing and bioinformatics analysis.** The preparation for ChIP sequencing samples was previously described[^7^](#_ENREF_7)^,^ [^8^](#_ENREF_8). Cells were treated with 25 mM glucose plus 100 nM insulin for 24 hours to activate STAT3 signaling. The chromatin was immunoprecipitated by antibody against anti-STAT3 (Cell Signaling Technology) or control IgG. DNA was then purified by QIAquick columns (QIAGEN) and quantified with Qubit Fluorometer (Thermo Fisher Scientific). ChIP DNA Libraries were prepared by VAHTS® Universal DNA Library Prep Kit for Illumina V3 (Vazyme) according to the manufacturer’s protocols. The sequence reads generated by Illumina sequencing were mapped to the human genome (mm10) using BWA algorithm, followed by peak calling with MACS2. The differential peaks in promoter regions were identified over the input control, then were analyzed for determining TF motifs with JASPAR database. And the differential peaks-related genes were analyzed for KEGG pathway analysis by DAVID.

**RNA isolation and quantitative real-time PCR analysis.** Tissues and cells were homogenized in TRIzol Reagent (Life Technologies) to extract total RNAs according to the manufacturer's protocol. Total RNAs were then reversely transcribed to cDNA using HiScript III RT SuperMix for qPCR (+gDNA wiper) (cat. R323-01, Vazyme). The resulting cDNA was subjected to real-time PCR with gene-specific primers in the presence of ChamQ SYBR qPCR Master Mix (High ROX Premixed) (cat. Q341-02, Vazyme) using StepOnePlus Real-Time PCR System (Applied Biosystems) as described previously[^6^](#_ENREF_6). The specificity of the PCR amplification was verified by analyzing the melting curve. Data were analyzed using the ΔΔCT threshold cycle method. The mRNA levels of genes were normalized to those of β-actin and presented as relative levels to control.

**Reference**

1. Gong Q, Hu Z, Zhang F, Cui A, Chen X, Jiang H, Gao J, Chen X, Han Y, Liang Q, Ye D, Shi L, Chin YE, Wang Y, Xiao H, Guo F, Liu Y, Zang M, Xu A and Li Y. Fibroblast growth factor 21 improves hepatic insulin sensitivity by inhibiting mammalian target of rapamycin complex 1 in mice. *Hepatology*. 2016;64:425-38.

2. Radaeva S, Jaruga B, Hong F, Kim WH, Fan S, Cai H, Strom S, Liu Y, El-Assal O and Gao B. Interferon-alpha activates multiple STAT signals and down-regulates c-Met in primary human hepatocytes. *Gastroenterology*. 2002;122:1020-34.

3. Yao J, Wu D, Zhang C, Yan T, Zhao Y, Shen H, Xue K, Huang X, Wang Z and Qiu Y. Macrophage IRX3 promotes diet-induced obesity and metabolic inflammation. *Nat Immunol*. 2021;22:1268-1279.

4. Ran FA, Hsu PD, Wright J, Agarwala V, Scott DA and Zhang F. Genome engineering using the CRISPR-Cas9 system. *Nat Protoc*. 2013;8:2281-2308.

5. Li Y, Xu S, Mihaylova MM, Zheng B, Hou X, Jiang B, Park O, Luo Z, Lefai E, Shyy JY, Gao B, Wierzbicki M, Verbeuren TJ, Shaw RJ, Cohen RA and Zang M. AMPK phosphorylates and inhibits SREBP activity to attenuate hepatic steatosis and atherosclerosis in diet-induced insulin-resistant mice. *Cell Metab*. 2011;13:376-388.

6. Chen X, Zhang F, Gong Q, Cui A, Zhuo S, Hu Z, Han Y, Gao J, Sun Y, Liu Z, Yang Z, Le Y, Gao X, Dong LQ, Gao X and Li Y. Hepatic ATF6 Increases Fatty Acid Oxidation to Attenuate Hepatic Steatosis in Mice Through Peroxisome Proliferator-Activated Receptor alpha. *Diabetes*. 2016;65:1904-15.

7. Li Y, Wong K, Walsh K, Gao B and Zang M. Retinoic acid receptor beta stimulates hepatic induction of fibroblast growth factor 21 to promote fatty acid oxidation and control whole-body energy homeostasis in mice. *J Biol Chem*. 2013;288:10490-504.

8. Liu Y, Su W, Liu Z, Hu Z, Shen J, Zheng Z, Ding D, Huang W, Li W, Cai G, Wei S, Li N, Fang X, Li H, Qin J, Zhang H, Xiao Y, Bi Y, Cui A, Zhang C and Li Y. Macrophage CREBZF Orchestrates Inflammatory Response to Potentiate Insulin Resistance and Type 2 Diabetes. *Adv Sci (Weinh)*. 2024;11:e2306685.
