# Supplementary figures and images for: Hepatoma‐Derived Growth Factor Coordinates STAT3 Pathway and Exosome‐Mediated Intrahepatic Crosstalk to Control Hepatic Steatosis and MASLD
Source: Adv Sci (Weinh). 2026 May 1;13(42):e23964. doi: 10.1002/advs.202523964 (PMC13335579; doi:10.1002/advs.202523964)

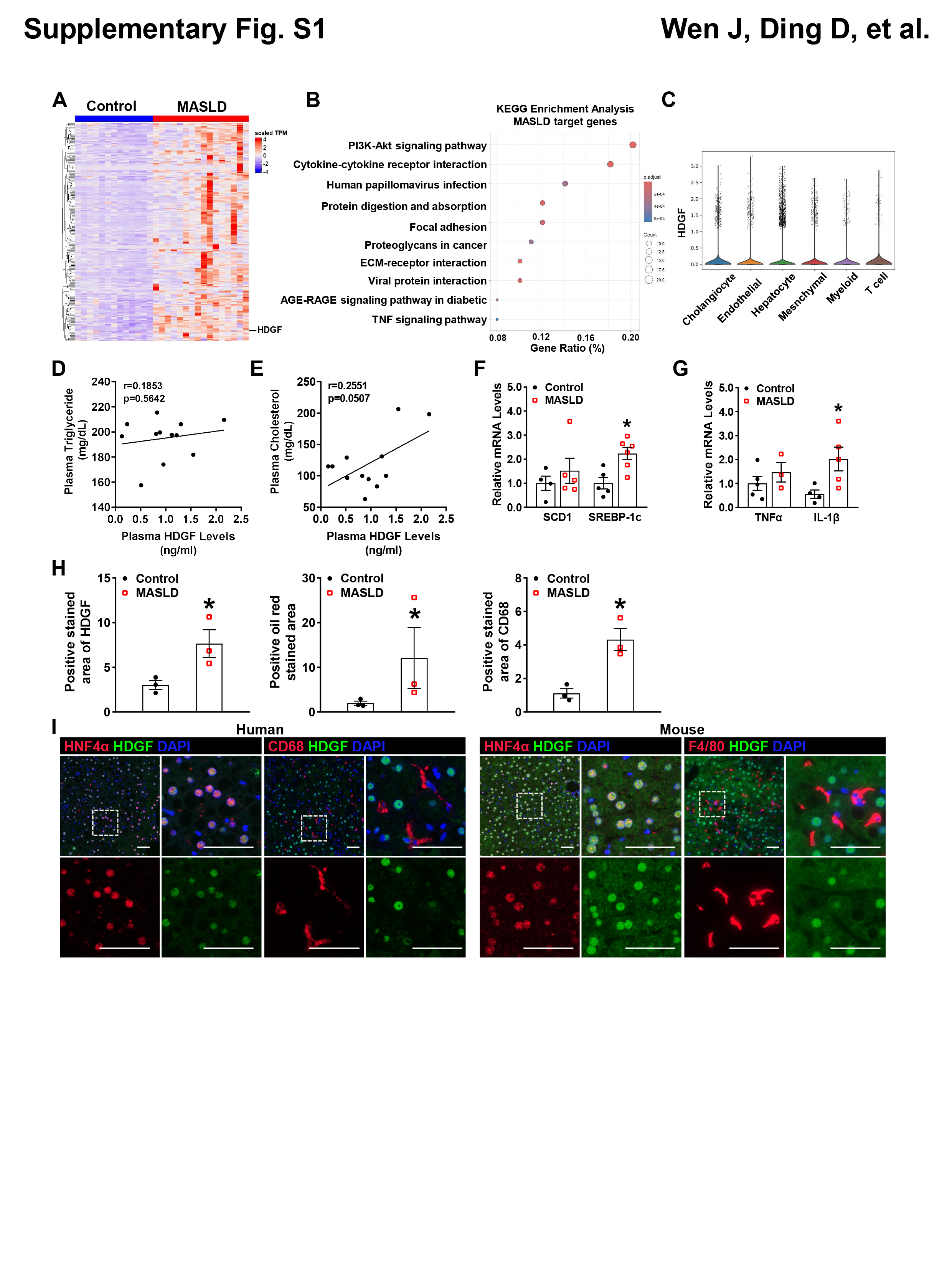

Supplement: Supplementary file 2 — Supporting File 2: advs75467‐sup‐0002‐FigureS1‐S7.zip. [file ADVS-13-e23964-s003.zip › Supplemental Figure-1.tif]

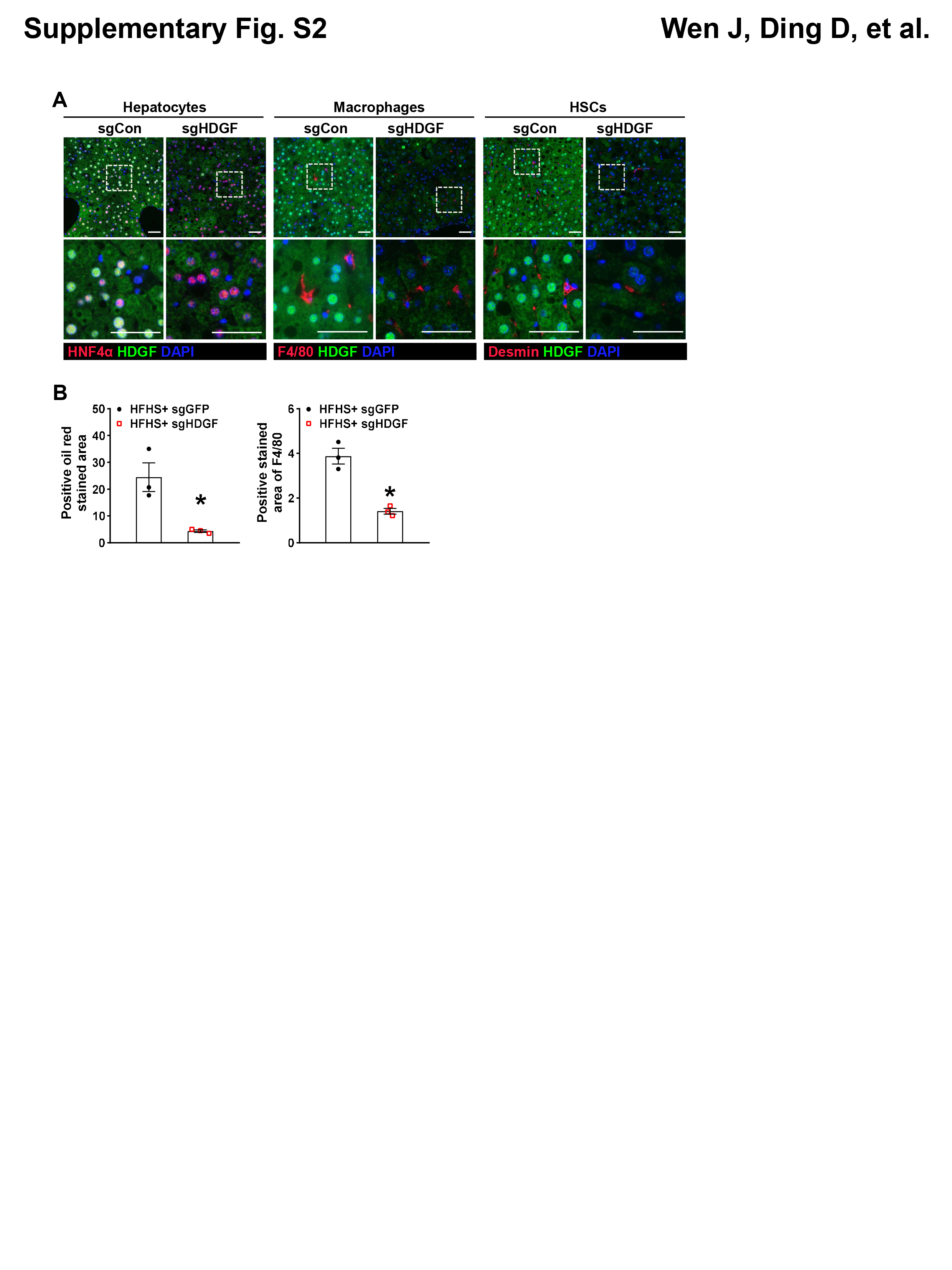

Supplement: Supplementary file 2 — Supporting File 2: advs75467‐sup‐0002‐FigureS1‐S7.zip. [file ADVS-13-e23964-s003.zip › Supplemental Figure-2.tif]

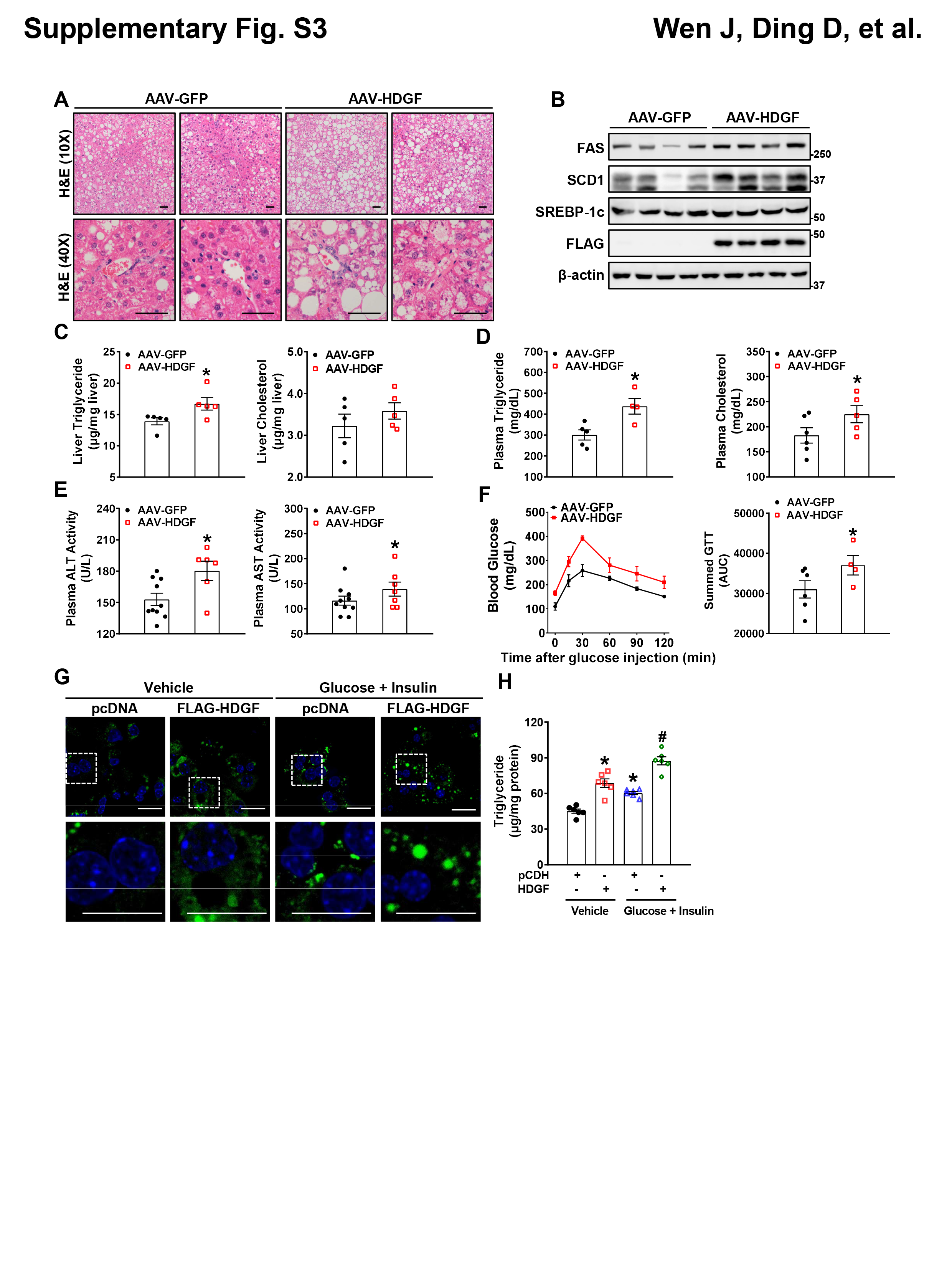

Supplement: Supplementary file 2 — Supporting File 2: advs75467‐sup‐0002‐FigureS1‐S7.zip. [file ADVS-13-e23964-s003.zip › Supplemental Figure-3.tif]

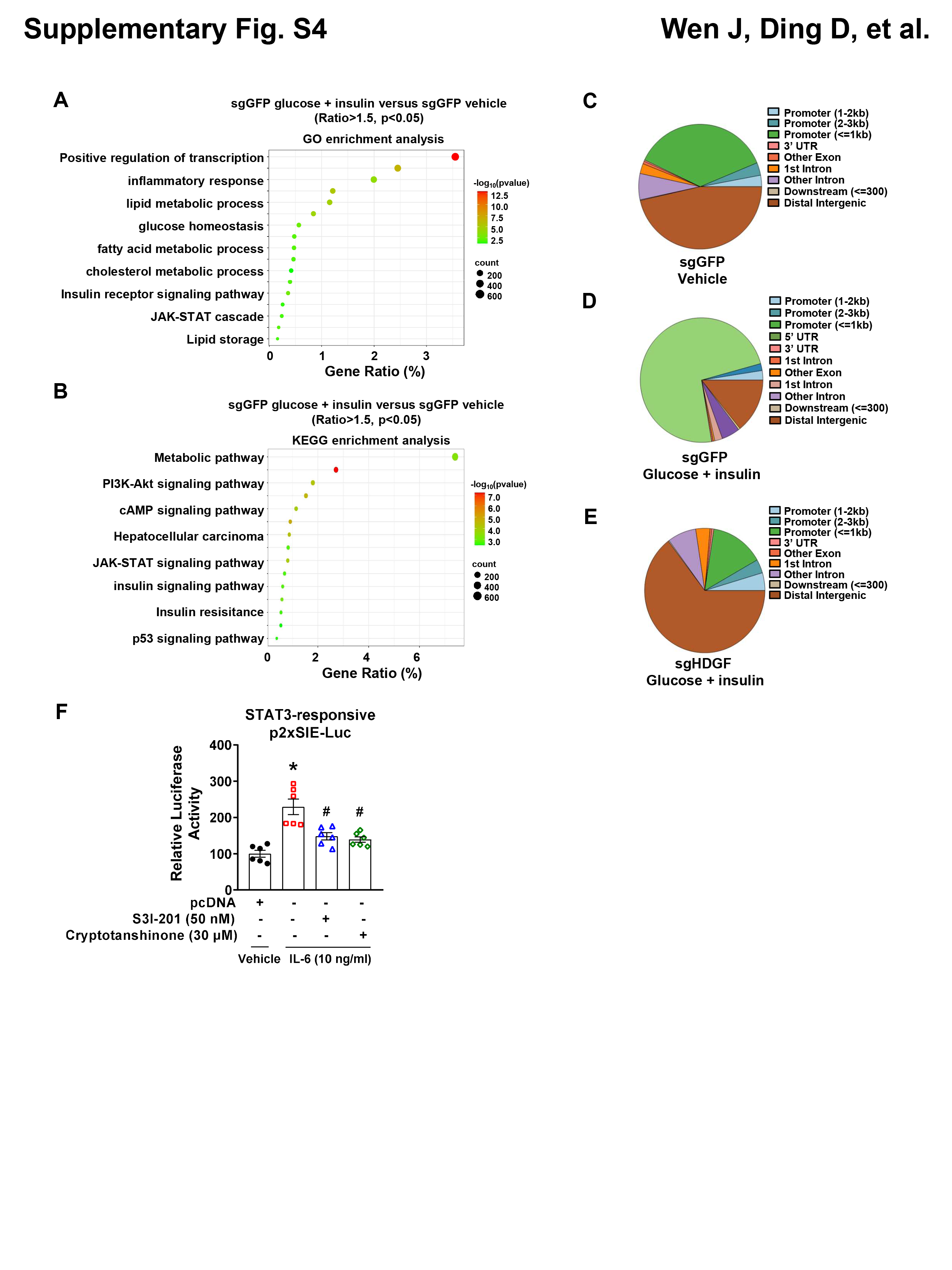

Supplement: Supplementary file 2 — Supporting File 2: advs75467‐sup‐0002‐FigureS1‐S7.zip. [file ADVS-13-e23964-s003.zip › Supplemental Figure-4.tif]

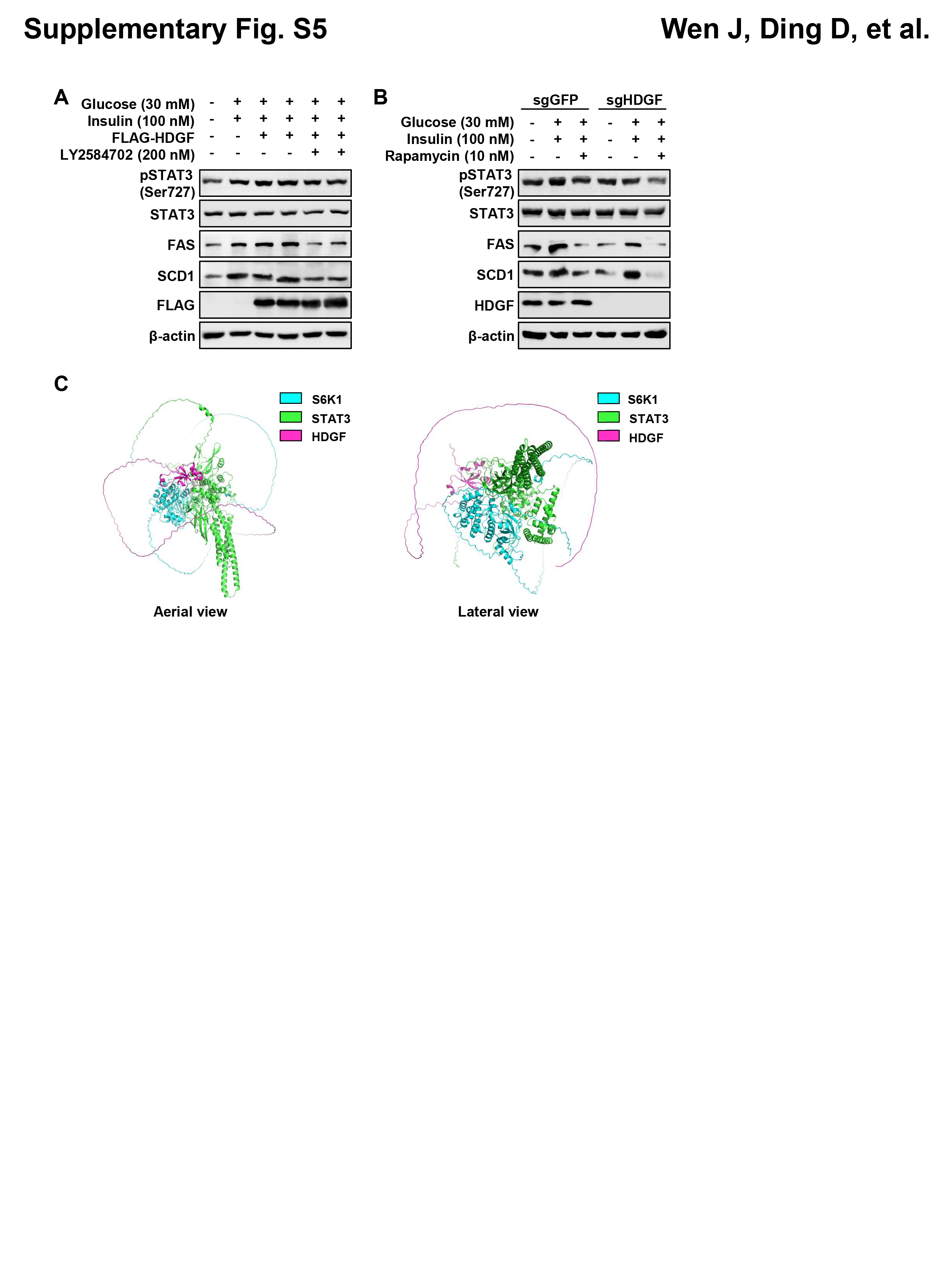

Supplement: Supplementary file 2 — Supporting File 2: advs75467‐sup‐0002‐FigureS1‐S7.zip. [file ADVS-13-e23964-s003.zip › Supplemental Figure-5.tif]

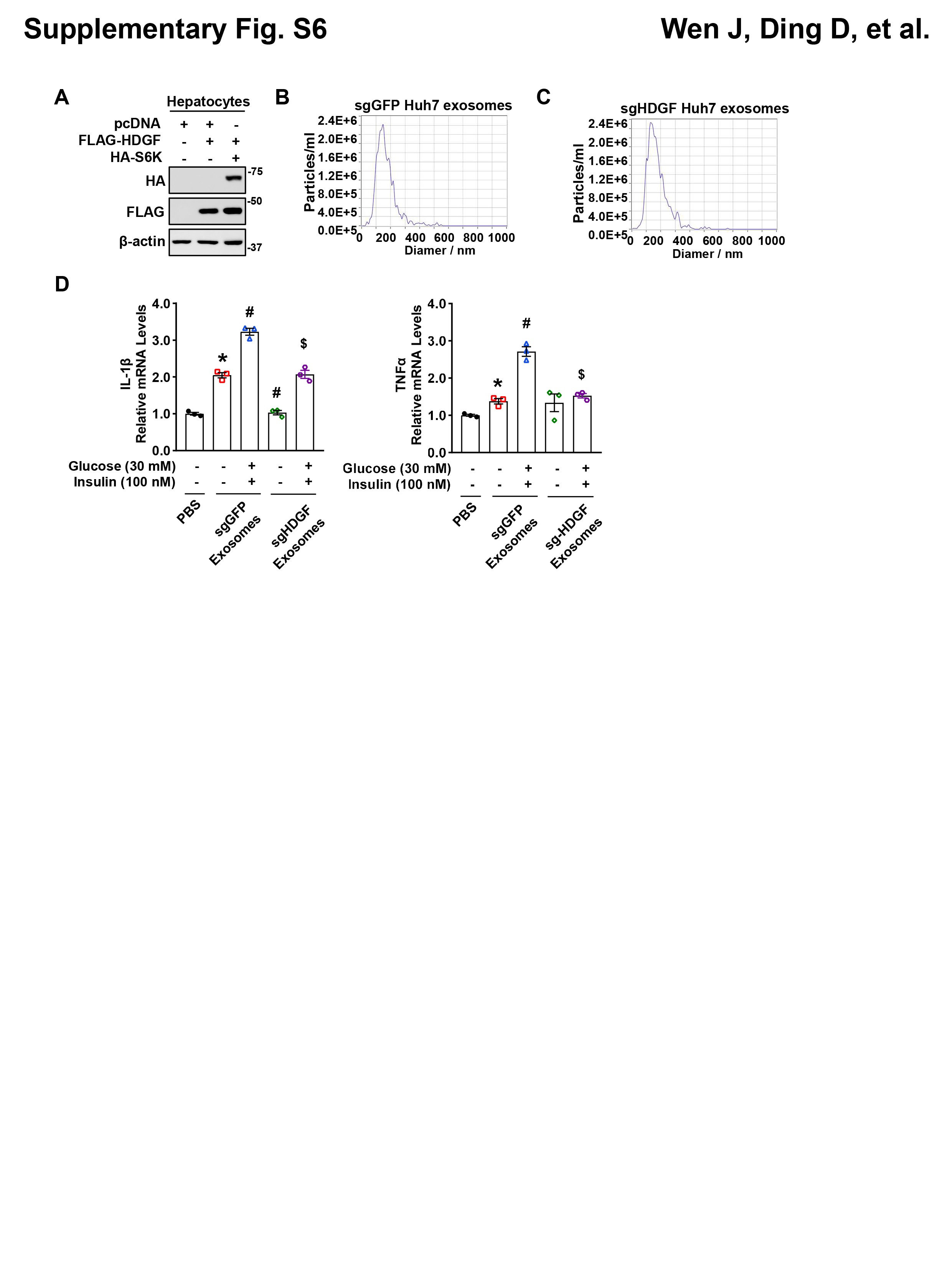

Supplement: Supplementary file 2 — Supporting File 2: advs75467‐sup‐0002‐FigureS1‐S7.zip. [file ADVS-13-e23964-s003.zip › Supplemental Figure-6.tif]

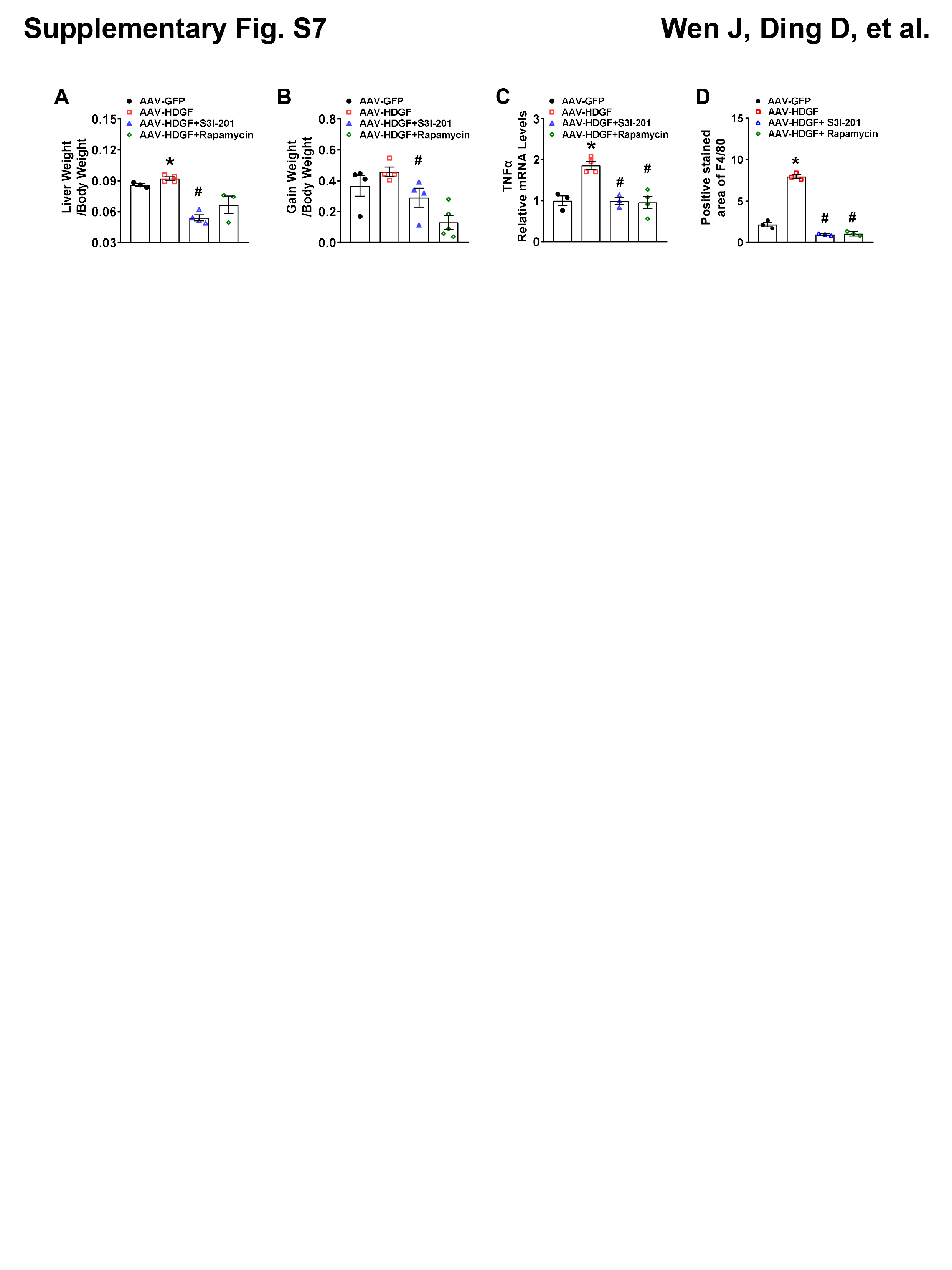

Supplement: Supplementary file 2 — Supporting File 2: advs75467‐sup‐0002‐FigureS1‐S7.zip. [file ADVS-13-e23964-s003.zip › Supplemental Figure-7.tif]
