# Supplementary material for: Hepatoma‐Derived Growth Factor Coordinates STAT3 Pathway and Exosome‐Mediated Intrahepatic Crosstalk to Control Hepatic Steatosis and MASLD
Source: Adv Sci (Weinh). 2026 May 1;13(42):e23964. doi: 10.1002/advs.202523964 (PMC13335579; doi:10.1002/advs.202523964)
Supplement: Supplementary file 3 — Supporting File 3: advs75467‐sup‐0003‐TableS1.docx. [file ADVS-13-e23964-s002.docx]

**Table S1. Quantitative RT-PCR primers**

| β-Actin | mouse | CCACAGCTGAGAGGGAAATC | AAGGAAGGCTGGAAAAGAGC |
| --- | --- | --- | --- |
| IL-1β | mouse | TTCGTGAATGAGCAGACAGC | GGTTTCTTGTGACCCTGAGC |
| IL-6 | mouse | AGTTGCCTTCTTGGGACTGA | TCCACGATTTCCCAGAGAAC |
| TNFα | mouse | CGTCAGCCGATTTGCTATCT | CGGACTCCGCAAAGTCTAAG |
| SREBP-1c | mouse | CCATCGACTACATCCGCTTC | GCCCTCCATAGACACATCTG |
| FAS | mouse | GCTGCGGAAACTTCAGGAAAT | AGAGACGTGTCACTCCTGGACTT |
| ACC1 | mouse | CTGTACGGGATCATACTGGTTC | ACAGTGGACAGAATTGAGGG |
| SCD1 | mouse | CCTCTGGAGGAGGAACGCTAAT | CGTGCCTTGTAAGTTCTGTG |
| HMGCR | mouse | AGAGCGAGTGCATTAGCAAAG | GATTGCCATTCCACGAGCTAT |
| HDGF | mouse | CCGGATTGATGAGATGCCTGA | GGCCTTGACTGTAGGGTTGTT |
| β-Actin | human | GATGAGATTGGCATGGCTTT | GTCACCTTCACCGTTCCAGT |
| HDGF | human | CTCTTCCCTTACGAGGAATCCA | CCTTGACAGTAGGGTTGTTCTC |
| IL-1β | human | TACTGGTCTCAGCGTCTCCA | CCGTCATGGGGAAGTCACTC |
| IL-6 | human | AGCAGGCACCCCAGTTAATC | TTTGCAGTGGAGGGATGCTC |
| TNFα | human | GAGGCCAAGCCCTGGTATG | CGGGCCGATTGATCTCAGC |
| SCD1 | human | GGGATCCTTCAGCACAGGAAT | GTCTGGCTAGTTATCCACCGCT |
| SREBP-1c | human | GCGCCTTGACAGGTGAAGTC | GCCAGGGAAGTCACTGTCTTG |
